# Epidemiologic Characteristics of Acute Kidney Injury During Cisplatin Infusions in Children Treated for Cancer

**DOI:** 10.1001/jamanetworkopen.2020.3639

**Published:** 2020-05-08

**Authors:** Kelly R. McMahon, Shahrad Rod Rassekh, Kirk R. Schultz, Tom Blydt-Hansen, Geoffrey D. E. Cuvelier, Cherry Mammen, Maury Pinsk, Bruce C. Carleton, Ross T. Tsuyuki, Colin J. D. Ross, Ana Palijan, Louis Huynh, Mariya Yordanova, Frédérik Crépeau-Hubert, Stella Wang, Debbie Boyko, Michael Zappitelli

**Affiliations:** 1 Research Institute of the McGill University Health Centre, Montreal Children’s Hospital, Division of Nephrology, Department of Pediatrics, McGill University Health Centre, Montreal, Quebec, Canada; 2 Division of Experimental Medicine, Faculty of Medicine, McGill University, Montreal, Quebec, Canada; 3 British Columbia Children’s Hospital, Division of Hematology/Oncology/Bone Marrow Transplantation, Department of Pediatrics, The University of British Columbia, Vancouver, British Columbia, Canada; 4 British Columbia Children’s Hospital, Division of Pediatric Nephrology, Department of Pediatrics, The University of British Columbia, Vancouver, British Columbia, Canada; 5 CancerCare Manitoba, Division of Pediatric Oncology-Hematology-BMT, Department of Pediatrics and Child Health, University of Manitoba, Winnipeg, Manitoba, Canada; 6 Section of Pediatric Nephrology, Department of Pediatrics and Child Health, University of Manitoba, Winnipeg, Manitoba, Canada; 7 BC Children’s Hospital Research Institute, Department of Pediatrics, Division of Translational Therapeutics, The University of British Columbia, Vancouver, British Columbia, Canada; 8 Epidemiology Coordinating and Research Centre, Department of Pharmacology, Faculty of Medicine and Dentistry, University of Alberta, Edmonton, Alberta, Canada; 9 Faculty of Pharmaceutical Sciences, The University of British Columbia, Vancouver, British Columbia, Canada; 10 Faculty of Health Sciences, Queen’s University, Kingston, Ontario, Canada; 11 Peter Gilgan Centre For Research and Learning, Toronto, Ontario, Canada; 12 Department of Pediatrics, Division of Pediatric Nephrology, Montreal Children’s Hospital, McGill University Health Centre, Montreal, Quebec, Canada; 13 Now with Toronto Hospital for Sick Children, Department of Pediatrics, Division of Nephrology, University of Toronto, Toronto, Ontario, Canada

## Abstract

**Importance:**

Few multicenter pediatric studies have comprehensively described the epidemiologic characteristics of cisplatin-associated acute kidney injury using standardized definitions.

**Objective:**

To examine the rate of and risk factors associated with acute kidney injury among children receiving cisplatin infusions.

**Design, Setting, and Participants:**

This prospective cohort study examined children (aged <18 years) recruited from May 23, 2013, to March 31, 2017, at 12 Canadian pediatric academic health centers who were receiving 1 or more cisplatin infusion. Children whose estimated or measured glomerular filtration rate (GFR) was less than 30 mL/min/1.73 m^2^ or who had received a kidney transplant were excluded. Data analysis was performed from January 3, 2018, to September 20, 2019.

**Exposures:**

Cisplatin infusions.

**Main Outcomes and Measures:**

The primary outcome was acute kidney injury during cisplatin infusion, defined using a Kidney Disease: Improving Global Outcomes serum creatinine criteria–based definition (stage 1 or higher). The secondary outcome was acute kidney injury defined by electrolyte criteria from the National Cancer Institute Common Terminology Criteria for Adverse Events (grade 1 or higher). Assessments occurred at early (first or second cycle) and late (last or second to last cycle) cisplatin infusions.

**Results:**

A total of 159 children (mean [SD] age at early cisplatin infusion, 7.2 [5.3] years; 80 [50%] male) participated. The most common diagnoses were central nervous system tumors (58 [36%]), neuroblastoma (43 [27%]), and osteosarcoma (33 [21%]). Acute kidney injury (by serum creatinine level increase) occurred in 48 of 159 patients (30%) at early cisplatin infusions and 23 of 143 patients (16%) at late cisplatin infusions. Acute kidney injury (by electrolyte abnormalities) occurred in 106 of 159 patients (67%) at early cisplatin infusion and 100 of 143 patients (70%) at late cisplatin infusions. Neuroblastoma diagnosis and higher precisplatin GFR were independently associated with acute kidney injury (serum creatinine level increase) at early cisplatin infusions (adjusted odds ratio [aOR] for neuroblastoma vs other, 3.25; 95% CI, 1.18-8.95; aOR for GFR, 1.01; 95% CI, 1.00-1.03) and late cisplatin infusions (aOR for neuroblastoma vs other, 6.85; 95% CI, 1.23-38.0; aOR for GFR, 1.01; 95% CI, 1.00-1.03). Higher cisplatin infusion dose was also independently associated with acute kidney injury (serum creatinine level increase) at later cisplatin infusions (aOR, 1.05; 95% CI, 1.01-1.10).

**Conclusions and Relevance:**

The findings suggest that acute kidney injury is common among children receiving cisplatin infusions and that rate and risk factors differ at earlier vs later infusions. These results may help with risk stratification with a goal of risk reduction.

## Key Points

**Question:**

What are the characteristics and risk factors associated with acute kidney injury among children receiving cisplatin infusions?

**Findings:**

In this cohort study of 159 cisplatin-treated children, 16% to 30% developed acute kidney injury during earlier (first or second cycle) and later (second to last or last cycle) cisplatin infusion treatment and 70% developed electrolyte disturbances. Cancer type and preinfusion kidney function were significantly associated with acute kidney injury risk at earlier and later infusions, and cisplatin dose was associated with acute kidney injury risk at later infusions.

**Meaning:**

The findings suggest that acute kidney injury is common during cisplatin infusions and that rate and risk factors differ at earlier vs later infusions.

## Teaser

This cohort study examines the rate of and risk factors associated with acute kidney injury in children treatment who receive cisplatin infusions.

## Introduction

Nephrotoxic medications are associated with acute kidney injury (AKI) in hospitalized children.^1,2^ Cisplatin, a chemotherapy commonly used to treat pediatric solid tumors (brain tumors, neuroblastoma, hepatoblastoma, and osteosarcoma), is associated with AKI via inflammation, mitochondrial and microvascular dysfunction, and oxidative stress.^3,4,5,6,7,8,9^ Children treated with cisplatin may have an increased risk of later chronic kidney disease (CKD) or hypertension, possibly because of repeated cisplatin-associated AKI episodes.^10^

Several knowledge gaps remain about cisplatin-associated AKI epidemiologic characteristics. Cisplatin-associated AKI is characterized by electrolyte wasting and/or polyuria with or without a serum creatinine (SCr) level increase.^11,12,13,14,15^ Depending on definitions used, the cisplatin-associated AKI rate is 27% to 100%.^11,12,13,14,15,16,17,18,19,20^ Few studies have described cisplatin-associated AKI using the Kidney Disease: Improving Global Outcomes (KDIGO) definition.^21^ However, this definition (mainly SCr based) may not adequately portray the cisplatin-associated AKI phenotype, which includes electrolyte disturbances (hypomagnesemia, hypokalemia, hypophosphatemia, and/or kidney tubular acidosis). A cisplatin-specific AKI definition that includes electrolyte abnormalities may more accurately reflect cisplatin-associated AKI phenotype associations with outcomes. Evidence on factors associated with AKI among children receiving cisplatin is limited; past studies^12,13,17,19,22^ were small, were single center, and used nonstandardized AKI definitions. Accurate description of cisplatin-associated AKI is needed for effective AKI risk stratification and design of AKI interventional trials to mitigate risk.

Inherent to the primary aim of the Applying Biomarkers to Minimize Long-Term Effects of Childhood/Adolescent Cancer Treatment (ABLE) Nephrotoxicity study, we aimed to describe the AKI phenotype (rate, severity, and duration) and risk factors associated with AKI during individual cisplatin infusions (early and later in cancer treatment) in children using standardized SCr-based AKI (SCr-AKI) and electrolyte-based AKI (eAKI) definitions.^23^ We hypothesized that AKI would be common during cisplatin infusions, that AKI would be more common and have unique risk factors at later vs earlier infusions, and that the phenotype would differ when AKI is defined using electrolytes.

## Methods

### Study Cohort

The ABLE Nephrotoxicity study was a multicenter, prospective cohort study of children treated with cisplatin who were recruited from May 23, 2013, to March 31, 2017, at 12 Canadian pediatric oncology centers.^23^ Data analysis was performed from January 3, 2018, to September 20, 2019. Inclusion criteria were age younger than 18 years at cancer diagnosis and planned cisplatin treatment (cisplatin naive or received at most 1 cisplatin cycle). Exclusion criteria included kidney transplant or estimated or measured glomerular filtration rate (GFR) less than 30 mL/min/1.73 m^2^. Sites obtained research ethics board approval. Written informed consent (assent when appropriate) was obtained before enrollment. This study followed the Strengthening the Reporting of Observational Studies in Epidemiology (STROBE) reporting guideline.^24^

### Study Procedure 

Children are typically hospitalized to receive cisplatin. Most cisplatin protocols comprise 2 to 8 cycles given every 3 to 6 weeks; dosages vary by tumor type (eg, single high-dose bolus [70-100 mg/m^2^] vs 2-5 days of low-dose bolus [20-60 mg/m^2^ daily]).^25,26^ The first prospective cisplatin study visit occurred shortly after recruitment at the first or second cisplatin cycle of cancer treatment (early visit [EV]). The late visit (LV) occurred at the last or second to last cycle. At the EV and LV, blood (3 mL) and urine (30 mL) samples were collected on the same day but before cisplatin infusion (day 1), the morning after infusion (day 2), and just before hospital discharge (at most day 5 after infusion). The study protocol is shown in eFigure 1 in the Supplement.

### Specimens and Measurements

Sites centrifuged blood samples (1000*g* for 10 minutes at 21 °C). Serum and urine samples were frozen (–80 °C) until shipment to the McGill University Health Centre. Specimen collection, freezing, and shipment were monitored using Research Electronic Data Capture (REDCap).^27^ At the McGill University Health Centre, urine samples were thawed and processed (1000*g* for 10 minutes); serum and urine samples were measured for creatinine (isotope dilution mass spectrometry–traceable), potassium, magnesium, and phosphorus levels.

### Data Collection 

Data collection is summarized in eFigure 1 in the Supplement. Baseline (precisplatin) data were collected retrospectively (demographic characteristics [race/ethnicity, classified by site coordinators; options were investigator defined], cancer details, kidney history [hypertension, CKD, AKI, kidney anomalies, and family history]; medications taken 2 weeks before cisplatin treatment; precisplatin treatment measured GFR; and 24-hour creatinine clearance [CrCl]). Baseline GFR was ascertained using measured GFR if available or CrCl if unavailable; if both were unavailable, GFR was estimated using the CKD in Children (CKiD) equation (using the lowest 3-month precisplatin SCr level).^28^ Decreased GFR was defined using age-normative GFR thresholds.^29^ From 3 days before to 10 days after study cisplatin infusions, prospective data collection included the following: routinely measured SCr and electrolyte levels, nephrotoxic medications (acyclovir, aminoglycosides, amphotericin B, ifosfamide, nonsteroidal anti-inflammatory drugs, and acetylsalicylic acid), diuretics, and electrolyte supplements (magnesium, phosphorus, and potassium). Immediate precisplatin treatment estimated GFR was calculated using the CKiD equation.^28^ Data collected throughout cisplatin treatment included dialysis, intensive care unit (ICU) admissions, infections (positive blood culture results and documentation), and AKI episodes between study visits. Completed standardized case report forms were sent to the Epidemiology Coordinating and Research Centre (Edmonton) for data entry (REDCap) with regular data quality queries.^27^ Quality assurance measures were taken to avoid missing data.

### Outcomes

Two cisplatin-associated AKI definitions were ascertained. First, SCr-based AKI was defined and staged using a KDIGO SCr-based definition (primary outcome of stage 1 or higher AKI) (eTable 1 in the Supplement).^21^ Because cisplatin-associated AKI is nonoliguric, KDIGO urine output criteria were not used. KDIGO timing criteria (48 hours or 7 days) were not used because these were primarily designed for ICU populations.^21^ Similar to a previous study,^30^ a 10-day period was used to ascertain AKI, allowing for variable routine follow-up times of laboratory measurements after cisplatin treatment. For EV, the baseline SCr level was the lowest 3-month precisplatin treatment level; for participants with previous cisplatin exposure, 3-day pre-EV infusion values were also used. For LV, the baseline SCr level was the lowest 3-day pre-LV level. Peak SCr level was the highest SCr level 10 days after infusion. Severe SCr-based AKI was KDIGO stage 2 or higher.

Second, eAKI was defined using the National Cancer Institute Common Terminology Criteria for Adverse Events, version 4.0 (eTable 1 in the Supplement).^31^ The novel term *eAKI* was chosen as a secondary outcome to reflect that electrolyte losses occur because of kidney tubular injury. Using nadir electrolyte values within 10 days after infusion, eAKI was graded based on serum magnesium, potassium, and phosphorus concentrations (any grade 1 or higher abnormality) (eTable 1 in the Supplement).^31^ We explored a secondary eAKI definition in which oral or intravenous electrolyte (potassium, magnesium, or phosphorous) supplementation was also considered. Composite AKI was both SCr-based AKI and eAKI. SCr and electrolyte levels measured for routine care and the 3 study-specific measures were used to ascertain AKI at the EV and LV. We decided a priori to evaluate routine vs study-measured laboratory values and exclude measures with evident bias (addressing potential issues with serum storage effects).

We calculated the number of days with SCr-based AKI and eAKI, which was the number of days fulfilling SCr-based AKI and eAKI criteria, respectively, within the 10 days after cisplatin infusion. SCr-based AKI recovery at last SCr level measurement was defined as the last SCr level available in 10 days after infusion less than 1.5 times the baseline SCr level.^32,33^

At all EV and LV sample points, fractional excretion (FE) of electrolytes was calculated as follows^34^: [Urine_[Electrolyte]_ × SCr/Urine_[Creatinine]_ × Serum_[Electrolyte]_] × 100 = FE_[Electrolyte]_ (%) (Serum Magnesium ×0.7).

### Statistical Analysis

Sample size was predetermined based on a convenience sample to balance feasibility and power.^23^ Variables were compared between groups using distribution-appropriate tests (Mann-Whitney test, Wilcoxon signed-rank test, 2-tailed, unpaired *t* test, χ^2^ test, or Fisher exact test). Spearman correlation between routine and study-measured laboratory values was examined. Percent agreement between AKI definitions was evaluated. Undocumented events and data were considered absent (negative).

Variables associated with AKI were evaluated separately for EV and LV using multivariable logistic regression. Candidate risk factors were categorized as baseline (before cisplatin administration), immediately before or on the day of cisplatin administration, and after cisplatin administration. Purposeful selection and manual backward selection were used to select variables for regression models. Variable associations, correlations, and collinearity were assessed. On the basis of the literature and clinical rationale, we a priori forced age into models. Variables associated with AKI in univariable analyses (*P* < .10) were initially included in multivariable models. Models were reduced (*P* < .05, for variable retention) to obtain parsimonious models. Variable deletion was confirmed using the backward likelihood ratio test. In final multivariable models, variables not significantly associated with AKI (*P* > .05) but considered significant confounders (ie, elimination significantly affected other β coefficients) were retained. Model fit was assessed (Hosmer-Lemeshow goodness of fit and area under the receiver operating characteristic curve [AUC]). Odds ratios (ORs) with 95% CIs were calculated. Separate regression models were used for SCr-based AKI and SCr plus eAKI. A 2-tailed *P* < .05 was considered statistically significant unless stated otherwise. Analyses were performed using Stata software, version 12.1 (StataCorp).

## Results

### Study Cohort

 Twelve of the 17 Canadian pediatric cancer centers (71%) were represented. Of 209 eligible patients approached, 159 were enrolled (76% consent rate (mean [SD] age at early cisplatin infusion, 7.2 [5.3] years; 80 [50%] male) (Figure). Late visit data were not evaluable for 16 participants because the EV occurred at the last cisplatin cycle (Figure). Specimens were available for 158 patients (99%) at the EV; specimens from 129 of 143 (90%) were available at the LV (Figure). Sample collection was successful (EV, ≥92%; LV, ≥83%) (eTable 2 in the Supplement), and the rate of missing data was low (eTable 3 in the Supplement). At the EV, 91 of 159 participants (57%) were cisplatin naive. The LV occurred at a median of the third cisplatin cycle (interquartile range [IQR], second to fourth cycles); this was the last cycle for 119 of 143 participants (83%). Throughout cisplatin therapy, no participant received dialysis; 1 died of tumor progression.

**Figure.**
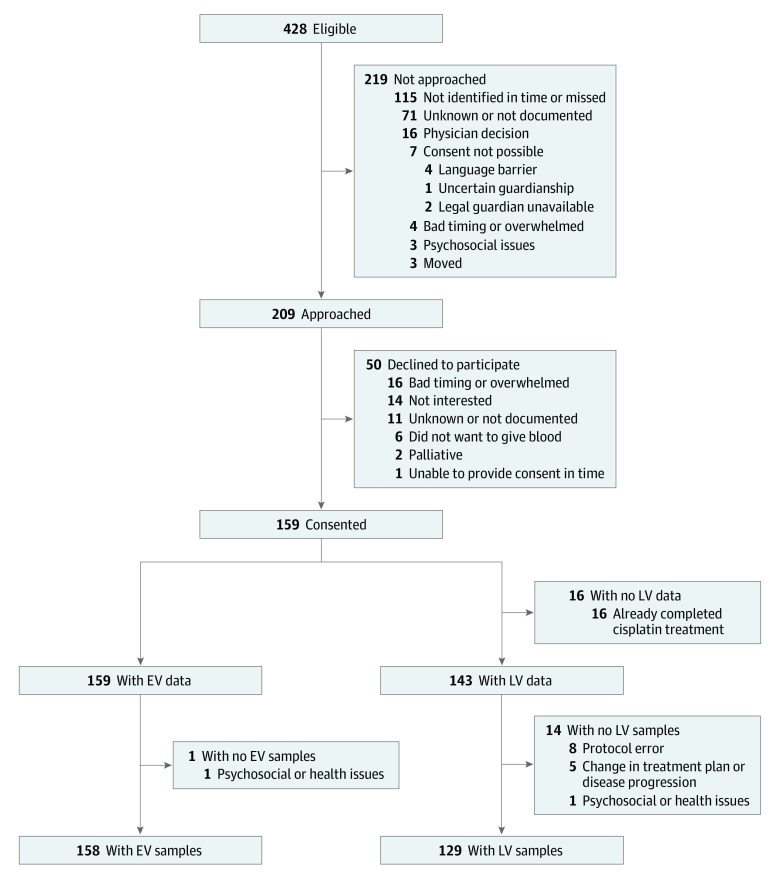
Flow Diagram of Study Participation and Data and Specimen Collection at Early and Late Cisplatin Study Visits The study flow diagram outlines the number of patients identified, recruited, and with available data and specimens at each cisplatin study visit (early visit [EV] and late visit [LV]). The top box (number eligible) refers to patients receiving cisplatin during the study activation period (at 6 sites, total number of patients receiving cisplatin may not be completely accurate). A total of 158 study participants had EV samples collected (blood or urine). A total of 129 study participants had LV blood or urine samples collected.

### Laboratory Measures 

Routinely measured SCr level correlated strongly with study-measured SCr level (Spearman ρ, >0.86) (eTable 4 in the Supplement). Routine phosphorus and magnesium level measurements correlated modestly with study-specific measures (phosphorus: ρ, >0.61; magnesium: ρ, >0.55). Routine potassium level measurements correlated variably with study-specific values (ρ, 0.33-0.64). Study-measured electrolyte levels were higher than routine measures, likely because of storage and hemolysis. Because the inclusion of study measures would tend to underestimate eAKI, we used all data available (worst of routine and study-specific measures).

### EV SCr-AKI

#### Baseline and AKI Characteristics

Of 159 participants, 117 (74%) were white. Central nervous system tumors (36%) and neuroblastoma (27%) were the most common diagnoses. At baseline, 8 participants (5%) had a history of hypertension, and 8 had a decreased GFR (measured or estimated) for age.

Forty-eight of the 159 participants (30%; 95% CI, 23%-37%) developed SCr-AKI at the EV; 12 of 159 (8%; 95% CI, 3%-12%) had severe SCr-AKI (stage 2 or higher). The median time with SCr-AKI was 2 days (IQR, 1-4 days).

Cancer type and a positive kidney medical history were associated with SCr-AKI (SCr-AKI: 42% CNS tumors, 38% neuroblastoma and 4% osteosarcoma, and 17% positive kidney medical history; no SCr-AKI: 34% CNS tumors, 23% neuroblastoma and 28% osteosarcoma, and 5% positive kidney medical history; other baseline characteristics were not associated with SCr-AKI) (Table 1). Two weeks before cisplatin treatment, nephrotoxic medication administration was uncommon (nonsteroidal anti-inflammatory drugs or acetylsalicylic acid [11%] and methotrexate [7%] were most common) (eTable 5 in the Supplement).

**Table 1.** Characteristics of Study Population at the EV and LV by SCr-AKI Status^a^

Characteristic	EV		LV	
	SCr-AKI (n = 48)	No SCr-AKI (n = 111)	SCr-AKI (n = 23)	No SCr-AKI (n = 120)
**Baseline (before first cisplatin infusion)**					
Male	23 (48)	57 (51)	8 (35)	61 (51)
White race/ethnicity	39 (81)	78 (70)	18 (78)	89 (74)
Cancer diagnosis				
CNS tumor^b^	20 (42)^c^	38 (34)	11 (48)^d^	44 (37)
Neuroblastoma	18 (38)	25 (23)	8 (35)	23 (19)
Osteosarcoma	2 (4)	31 (28)	0 (0)	33 (28)
Germ cell tumor	3 (6)	11 (10)	0 (0)	14 (12)
Hepatoblastoma	4 (8)	5 (5)	3 (13)	6 (5)
Other^e^	1 (2)	1 (1)	1 (4)	0 (0)
Cancer involves 1 or both kidneys	5 (10)	6 (5)	3 (13)	4 (3)
Kidney medical history^f^	8 (17)^c^	5 (5)	1 (4)	10 (8)
Any nephrotoxic drug before first cisplatin infusion^g^	12 (25)	15 (14)	NA	NA
Vancomycin in 2 wk before cisplatin infusion	5 (10)	3 (3)	NA	NA
**Immediately before or on the day of EV or LV infusion**					
Age at EV or LV, median (IQR), y	2.7 (2.0-6.8)^d^	7.5 (3.1-13.4)	2.6 (2.2-4.7)^c^	7.7 (2.6-13.7)
Age <3 y	26 (54)^c^	26 (23)	13 (57)^c^	32 (27)
Cisplatin naive at the EV	31 (65)	60 (54)	NA	NA
Previsit eGFR, median (IQR), mL/min/1.73 m^2^^h^	166 (140-206)^d^	132 (115-155)	169 (156-202)^d^	133 (114-163)
Preinfusion serum level, mean (SD)^i^				
Phosphorus, mg/dL	4.8 (1.6)	4.6 (0.9)	4.3 (0.8)^c^	4.8 (0.9)^j^
Magnesium, mEq/L	1.6 (0.3)	1.6 (0.2)	1.5 (0.2)	1.6 (0.2)^j^
Last cisplatin cycle of treatment at LV	NA	NA	19 (83)	100 (83)
Cumulative cisplatin dose before the EV or LV, median (IQR), mg/m^2^	82 (77-127)^k^	109 (77-120)^k^	168 (154-204)^c^	224 (167-353)
EV or LV cisplatin infusion dose, median (IQR), mg/m^2^	65 (50-77)	59 (49-74)	75 (49-80)^c^	58 (37-71)
Before EV or LV				
Infection	6 (13)	14 (13)	12 (52)	38 (32)
PICU admission	1 (2)	1 (1)	4 (17)	9 (8)
SCr-AKI episode^l^	11 (23)^c^	8 (7)	15 (65)	52 (43)
Between EV and LV visits, median (IQR), d	NA	NA	42 (29-64)^c^	69 (42-118)
Concurrent nephrotoxins at EV or LV^m^	3 (6)	20 (18)	2 (9)	20 (17)
Cancer treatment				
EV or LV total cisplatin cycle dose, median (IQR), mg/m^2^	100 (77-175)	109 (77-122)	83 (76-155)	100 (75-120)
Any flank, whole abdomen, pelvic, or total body radiotherapy planned	10 (21)	12 (11)	3 (13)	12 (10)
After cisplatin				
EV or LV length of stay, median (IQR), d	5 (4-24)^d^	4 (3-5)	4 (3-11)^c^	4 (2-5)
Nephrotoxins in 10 d after EV or LV^n^	6 (13)	7 (6)	1 (4)	10 (8)

Abbreviations: AKI, acute kidney injury; CNS, central nervous system; eGFR, estimated glomerular filtration rate; EV, early visit; IQR, interquartile range; LV, late visit; NA, not applicable; PICU, pediatric intensive care unit; SCr-AKI, serum creatinine–based AKI.

SI conversion factors: To convert magnesium to millimoles per liter, multiply by 0.5; phosphorus to millimoles per liter, multiply by 0.323.

^a^ Data are presented as number (percentage) of patients unless otherwise indicated. Percentages are based on the total for each column.

^b^ Astrocytoma (n = 3), choroid plexus tumor (n = 2), ependymoma (n = 1), medulloblastoma (n = 39), primitive neuroectodermal tumor (n = 7), and atypical teratoid or rhabdoid tumor (n = 6).

^c^ Significant difference between AKI and non-AKI groups for that time (*P* < .05).

^d^ Significant difference between AKI and non-AKI groups for that time (*P* < .001).

^e^ Lymphoma and nasopharyngeal carcinoma.

^f^ Hypertension, treatment with antihypertensives, family history of kidney disease, chronic kidney disease, dialysis, congenital kidney anomaly, kidney stones, vesicoureteral reflux, urinary tract infection and/or serum electrolyte abnormality requiring treatment, or AKI.

^g^ Receipt of acyclovir, amphotericin, aminoglycosides (gentamycin, tobramycin, or amikacin), vancomycin, angiotensin-converting enzyme inhibitor, ganciclovir, valganciclovir, ifosfamide, or methotrexate in the 2 weeks before cisplatin infusion.

^h^ Calculated using the updated Chronic Kidney Disease in Children equation.

^i^ Preinfusion electrolyte levels were determined by using the study-measured electrolyte (measured before or on day of cisplatin infusion); if unavailable, the most recent available routine electrolyte value was used. For the LV non-AKI group, n = 118 (preinfusion serum phosphorous and magnesium values not available for 2).

^j^ N = 118 (preinfusion electrolyte value not available for 2).

^k^ Data for 17 patients with SCr-AKI and 51 without (none were cisplatin naive at the EV).

^l^ All routine and study-measured SCr values measured before cisplatin infusion at the EV or LV were used. Peak SCr level was the highest SCr level occurring before the EV and LV infusion but occurring after the baseline SCr measurement. For SCr-AKI before the EV, the baseline SCr level was the lowest routine value in the 3 months before cisplatin initiation. For AKI between the EV and LV, the baseline SCr level was the lowest routine or study-measured value in 3 months before the first cisplatin infusion.

^m^ Acyclovir, amphotericin, aminoglycosides (gentamycin, tobramycin, or amikacin), ifosfamide, or chemotherapy protocol indicates that a nephrotoxin (aldesleukin, busulfan, carboplatin, dinutuximab, gemcitabine, ifosfamide, lomustine, melphalan, methotrexate, radiotherapy, rituximab, stem cell transplant, or temsirolimus) was given within 24 hours of cisplatin infusion.

^n^ Acyclovir, amphotericin, aminoglycosides (gentamycin, tobramycin, or amikacin), and/or ifosfamide.

#### EV and Cisplatin Treatment Characteristics

Age younger than 3 years (eFigure 2 in the Supplement) and higher previsit estimated GFR were associated with SCr-AKI at the EV (SCr-AKI: 54% aged <3 years and median eGFR of 166 mL/min/1.73 m^2^; no SCr-AKI: 23% aged <3 years and median eGFR of 132 mL/min/1.73 m^2^) (Table 1). Past SCr-AKI was more common in participants who did vs did not develop SCr-AKI (SCr-AKI: 23% with previous AKI; no SCr-AKI: 7% with previous AKI); ICU admission, infections, concurrent nephrotoxins, radiotherapy (planned in treatment protocol or given), and total cisplatin cycle dose did not differ by SCr-AKI status (Table 1). Participants with SCr-AKI (vs without) had a longer hospital stay (median of 5 days in patients with SCr-AKI and median of 4 days in those without SCr-AKI; Table 1).

### LV SCr-AKI

#### Baseline and AKI Characteristics

SCr-AKI at the LV was found in 23 of 143 patients (16%; 95% CI, 10%-22%); 7 of 143 (5%; 95% CI, 1%-8%) had severe SCr-AKI (stage 2 or higher) (Table 2). The median time with SCr-AKI was 1 day (IQR, 1-3 days). Cancer type was significantly associated with SCr-AKI (Table 1).

**Table 2.** Rate of SCr-AKI, eAKI, and SCr Plus eAKI and Staging or Grading at the EV and/or LV Using 3 Definitions

Variable	Children, No. (%) [95% CI]		
	SCr-AKI	eAKI	SCr plus eAKI
**At EV**			
Total	48/159 (30) [23-37]	106/159 (67) [59-74]	32/159 (20) [14-26]
Stage or grade			
0	111/159 (70) [63-77]	53/159 (33) [26-41]	NA
1	36/159 (23) [16-29]	71/159 (45) [37-52]	NA
2	10/159 (6) [2-10]	17/159 (11) [6-16]	NA
3	2/159 (1) [0-3]	16/159 (10) [5-15]	NA
4	NA	2/159 (1) [0-3]	NA
**At LV**			
Total	23/143 (16) [10-22]	100/143 (70) [62-78]	16/143 (11) [6-16]
Stage or grade			
0	120/143 (84) [78-90]	43/143 (30) [22-38]	NA
1	16/143 (11) [6-16]	76/143 (53) [45-61]	NA
2	5/143 (3) [4-7]	12/143 (8) [4-13]	NA
3	2/143 (1) [0-3]	10/143 (7) [3-11]	NA
4	NA	2/143 (1) [0-3]	NA
**At EV or LV**			
Total	59/159 (37) [30-45]	134/159 (84) [79-90]	41/159 (26) [19-33]
Stage or grade			
0	100/159 (63) [55-70]	25/159 (16) [10-21]	NA
1	42/159 (26) [19-33]	81/159 (51) [43-59]	NA
2	13/159 (8) [4-12]	25/159 (16) [10-21]	NA
3	4/159 (3) [0-5]	24/159 (15) [9-21]	NA
4	NA	4/159 (3) [0-5]	NA
**At EV and LV**			
Total	12/143 (8) [3-12]	72/143 (50) [42-59]	7/143 (5) [1-8]
Stage or grade			
0	131/143 (92) [87-96]	71/143 (50) [41-58]	NA
1	5/143 (3) [0-7]	36/143 (25) [18-32]	NA
2	6/143 (4) [1-8]	17/143 (12) [7-17]	NA
3	1/143 (1) [0-2]	16/143 (11) [6-16]	NA
4	NA	3/143 (2) [0-4]	NA

Abbreviations: AKI, acute kidney injury; eAKI, electrolyte-based AKI; EV, early visit; LV, late visit; NA, not applicable; SCr-AKI, serum creatinine–based AKI.

#### LV and Cisplatin Treatment Characteristics

 Age younger than 3 years and higher previsit estimated GFR were associated with SCr-AKI (57% aged <3 years and median GFR of 169 mL/min/1.73 m^2^ in those with SCr-AKI and 27% aged <3 years and median GFR of 133 mL/min/1.73 m^2^ in those without SCr-AKI; Table 1). Prior cumulative cisplatin dose was lower and cisplatin infusion dose was higher in participants with SCr-AKI (median prior dose of 168 mg/m^2^ in those with SCr-AKI and 224 mg/m^2^ in those without; median infusion dose of 75 mg/m^2^ in those with SCr-AKI and 58 mg/m^2^ in those without; Table 1). Time between the EV and LV was shorter in participants with (median of 42 days) vs without SCr-AKI (median of 69 days); ICU admission, infections, concurrent nephrotoxins, radiotherapy, and total cisplatin cycle dose were not associated with SCr-AKI (Table 1). Participants with SCr-AKI (vs without) had a longer hospital stay (median [IQR] of 4 [3-11] days in patients with SCr-AKI and 4 [2-5] days in those without SCr-AKI; Table 1).

### SCr Plus eAKI and eAKI

A total of 106 of the 159 participants (67%; 95% CI, 59%-74%) developed eAKI at the EV, and 100 of the 143 participants (70%; 95% CI, 62%-78%) developed eAKI at the LV. The median time with eAKI was 2 days (IQR, 1-3 days) at the EV and 2 days (IQR, 1-4 days) at the LV. Thirty-two of 159 patients (20%; 95% CI, 14%-26%) had SCr plus eAKI at the EV, and 16 of 143 (11%; 95% CI, 6%-16%) had SCr plus eAKI at the LV. eTable 6 and eTable 7 in the Supplement list the participant characteristics by eAKI and SCr plus eAKI status.

Hypophosphatemia mainly contributed to eAKI (Table 3). When including electrolyte supplementation criteria to define eAKI, the hypophosphatemia rate remained similar (EV: 57%; 95% CI, 49%-65%; LV: 56%; 95% CI, 48%-64%), the hypomagnesemia rate increased (EV: 78%; 95% CI, 71%-84%; LV: 83%; 95% CI, 77%-89%), and the hypokalemia rate increased (EV: 60%; 95% CI, 53%-68%; LV: 61%; 95% CI, 53%-69%). A total of 144 of 159 patients (91%; 95% CI, 86%-95%) had eAKI or received electrolyte supplements at the EV and 132 of 143 patients (92%; 95% CI, 88%-97%) had eAKI or received electrolyte supplements at the LV.

**Table 3.** Description of AKI Features at the EV and LV

Feature	Children, No. (%) [95% CI]	
	EV	LV
**SCr-AKI**		
Increase in SCr, median (IQR), %	74 (60-103) (n = 48)	71 (58-118) (n = 23)
Time to peak SCr of SCr-AKI, median (IQR), d	2 (1-5) (n = 48)	2 (1-4) (n = 23)
Time to SCr-AKI diagnosis, median (IQR), d	2 (1-5) (n = 48)	2 (1-4) (n = 23)
SCr-AKI recovery at last SCr measurement, No./total No. (%)^a^	22/48 (46)	13/23 (57)
Complete^b^	10/48 (21)	4/23 (17)
Partial^c^	12/48 (25)	9/23 (39)
None^d^	26/48 (54)	10/23 (43)
**eAKI**		
Time to nadir electrolyte of eAKI, median (IQR), d	2 (1-4) (n = 106)	2 (1-3) (n = 100)
Time to eAKI diagnosis, median (IQR), d	1.5 (1-3) (n = 106)	1 (1-2) (n = 100)
Hypophosphatemia	88/158 (56) [48-64]^e^	79/143 (55) [47-63]
Grade 0	70 (44) [36-52]	64 (45) [37-53]
Grade 1	62 (39) [32-47]	60 (42) [34-50]
Grade 3	21 (13) [8-19]	15 (10) [5-16]
Grade 3	5 (3) [4-6]	4 (3) [0-6]
Grade 4	0 (0) [0-0]	0 (0) [0-0]
Hypomagnesemia	42/158 (27) [20-34]^e^	56/143 (39) [31-47]
Grade 0	116 (73) [66-80]	87 (61) [53-69]
Grade 1	39 (25) [18-31]	49 (34) [26-42]
Grade 3	2 (1) [0-3]	7 (5) [1-8]
Grade 3	1 (1) [0-2]	0 (0) [0-0]
Grade 4	0 (0) [0-0]	0 (0) [0-0]
Hypokalemia	40/159 (25) [18-32]	25/143 (17) [11-24]
Grade 0	119 (75) [68-82]	118 (83) [76-89]
Grade 1	27 (17) [11-23]	14 (10) [5-15]
Grade 3	NA	NA
Grade 3	11 (7) [3-11]	9 (6) [2-10]
Grade 4	2 (1) [0-3]	2 (1) [0-3]

Abbreviations: AKI, acute kidney injury; eAKI, electrolyte-based AKI; EV, early visit; IQR, interquartile range; LV, late visit; NA, not applicable; SCr, serum creatinine; SCr-AKI, serum creatinine–based AKI.

^a^ Last SCr measurement available in the 10 days after cisplatin infusion less than 1.5 times baseline.

^b^ Last SCr measurement available in the 10 days after cisplatin infusion less than 1.2 times baseline.

^c^ Last SCr measurement available in the 10 days after cisplatin infusion 1.2 times or greater and less than 1.5 times baseline.

^d^ Last SCr measurement available in the 10 days after cisplatin infusion 1.5 times or greater than baseline.

^e^ Hypophosphatemia and hypomagnesemia status could not be assessed in 1 participant at the EV because no routine phosphorous or magnesium values were available and the study blood specimens were not successfully collected.

At the EV and LV, phosphorus concentrations decreased significantly from before infusion to after infusion and remained low or decreased at discharge (eFigure 3 in the Supplement). Serum magnesium and potassium concentrations increased from before infusion to after infusion and remained higher than before infusion at discharge (eFigure 3 in the Supplement). Fractional extraction of all electrolytes increased significantly from before infusion to after infusion (eTable 8 in the Supplement).

### Characteristics by Cancer Type

Participants with neuroblastoma and hepatoblastoma were younger and had high SCr-AKI rates (eTable 9 in the Supplement). Those with osteosarcoma and germ cell tumors had low SCr-AKI rates; none developed SCr-AKI at the LV (eTable 9 in the Supplement). Cisplatin dosage and administration schedule varied by cancer type (eTable 10 in the Supplement). Treatment protocols of participants with germ cell tumors, osteosarcoma, and hepatoblastoma included fewer potential nephrotoxins (eTable 10 in the Supplement). Treatment protocols of 2 of the 159 participants (1%) included amifostine, and treatment protocols of 13 of the 159 participants (8%) included mesna.

### AKI Definition and Visit Comparisons

Discordance between SCr-AKI and eAKI classifications was 57% at the EV and 64% at the LV. Of 48 participants with SCr-AKI at the EV, 12 of 41 (29%; 95% CI, 15%-44%) also had SCr-AKI at the LV; of 111 without SCr-AKI at the EV, 11 of 102 (11%; 95% CI: 5%-17%) had SCr-AKI at the LV. The median cisplatin infusion dose was lower at the LV (58 mg/m^2^; IQR, 37-74 mg/m^2^) compared with the EV (60 mg/m^2^; IQR, 50-75 mg/m^2^; *P* < .05).

### Factors Associated With AKI Risk

In multivariable analyses, central nervous system tumors, neuroblastoma, and higher pre-EV estimated GFR were associated with SCr-AKI at the EV (model AUC, 0.78; 95% CI, 0.69-0.86) (Table 4). For SCr plus eAKI at the EV, higher pre-EV estimated GFR and kidney medical history were associated with AKI (model AUC, 0.77; 95% CI, 0.68-0.86) (Table 4). Age younger than 3 years, neuroblastoma, higher LV cisplatin infusion dose, and higher pre-LV estimated GFR were associated with SCr-AKI at the LV (model AUC, 0.82; 95% CI, 0.74-0.91) (Table 4). Meaningful multivariable analyses for SCr plus eAKI at the LV were not feasible because of low event rate.

**Table 4.** Unadjusted and Adjusted Risk Factors for AKI at the EV and LV

Variable	Unadjusted OR (95% CI)	*P* Value	Adjusted OR (95% CI)	*P* Value
**Predicting SCr-AKI at the EV (n = 159)**				
Age <3 y at the EV^a^	3.86 (1.88-7.92)	<.001	1.76 (0.74-4.19)	.20
Cancer type				
Other cancers^b^^,^^c^	1 [Reference]	NA	1 [Reference]	NA
CNS tumors	2.53 (1.06-6.03)	.04	3.29 (1.18-9.18)	.02
Neuroblastoma	3.46 (1.39-8.60)	.01	3.25 (1.18-8.95)	.02
Pre-EV eGFR	1.02 (1.01-1.03)	<.001	1.01 (1.005-1.03)	.004
Concurrent nephrotoxins^d^	0.303 (0.086-1.07)	.07	0.38 (0.09-1.52)	.17
**Predicting SCr plus eAKI at the EV (n = 159)**				
Age <3 y at the EV^a^	2.98 (1.34-6.60)	.007	1.42 (0.53-3.84)	.49
Pre-EV eGFR	1.02 (1.01-1.03)	.001	1.01 (1.003-1.02)	.01
Kidney medical history^e^	5.65 (1.75-18.2)	.004	4.97 (1.37-18.0)	.02
Vancomycin in 2 weeks before the first cisplatin infusion^f^	4.39 (1.04-18.6)	.05	3.10 (0.58-16.7)	.19
**Predicting SCr-AKI at the LV (n = 143)**				
Age <3 y at the LV^a^	3.58 (1.43-8.96)	.007	1.73 (0.61-4.88)	.30
Cancer type				
Other cancers^c^^,^^g^	1 [Reference]	NA	1 [Reference]	NA
CNS tumors	3.31 (0.99-11.1)	.05	1.65 (0.41-6.60)	.48
Neuroblastoma	4.61 (1.26-16.8)	.02	6.85 (1.23-38)	.03
LV cisplatin infusion dose, mg/m^2^	1.03 (1.01-1.06)	.01	1.05 (1.01-1.10)	.03
Pre-LV eGFR	1.02 (1.01-1.03)	<.001	1.01 (1.002-1.03)	.02

Abbreviations: AKI, acute kidney injury; CNS, central nervous system; eAKI, electrolyte-based AKI; eGFR, estimated glomerular filtration rate; EV, early visit; LV, late visit; NA, not applicable; OR, odds ratio; SCr, serum creatinine; SCr-AKI, serum creatinine–based AKI.

^a^ Age was categorized as younger than 3 years because there was a clear increased AKI risk in this group (eFigure 2 in the Supplement) and to increase clinical usefulness. Age younger than 3 years was forced into all models.

^b^ Other cancer diagnoses include osteosarcoma, germ cell tumors, hepatoblastoma, lymphoma, and nasopharyngeal carcinoma.

^c^ On the basis of distribution, cancer type was expressed as 3 categories: CNS tumors, neuroblastoma, and other cancers (osteosarcoma, germ cell tumors, hepatoblastoma, and others).

^d^ Concurrent nephrotoxins was kept in the SCr-AKI EV model because it was an important confounder (caused a ≥20% change in the point estimate of other covariates).

^e^ Kidney medical history included hypertension, treatment with antihypertensives, family history of kidney disease, chronic kidney disease, dialysis, congenital kidney anomaly, kidney stones, vesicoureteral reflux, urinary tract infection, or AKI.

^f^ Vancomycin in the 2 weeks before the first cisplatin infusion was kept in the SCr plus eAKI EV model because it was an important confounder (caused a ≥20% change in the point estimate of other covariates).

^g^ Cancer types were 53 CNS tumors, 9 neuroblastoma, and 57 other cancers. Other cancer diagnoses include osteosarcoma (n = 33), germ cell tumors (n = 14), hepatoblastoma (n = 9), and others (n = 1 with nasopharyngeal carcinoma).

## Discussion

To our knowledge, this is the first multicenter, prospective, pediatric study using a KDIGO-based AKI definition to study cisplatin-associated AKI characteristics and risk factors. SCr-AKI was more common at the EV compared with the LV. Concordance between SCr-AKI and eAKI was poor; eAKI was more common than SCr-AKI.

The AKI definition affected the AKI rate. The SCr-AKI rate at the EV was comparable to previous studies (27%-75%), and the LV SCr-AKI rate was comparable to adult estimates.^11,14,16,20,30,35,36,37^ However, most studies^11,14^ evaluated SCr-AKI throughout chemotherapy (including multiple chemotherapies and cycles), whereas we evaluated 2 individual cisplatin cycles, which may be more relevant for individual patient care. Discordance between SCr-AKI and eAKI suggests that each provides distinct information; eAKI may more directly reflect tubular injury. SCr may be an inaccurate AKI marker in this setting because of low muscle mass. Other biomarkers (eg, cystatin C) should be evaluated. Fluids are commonly administered before cisplatin infusion, concurrently, and after infusion, which could dilute SCr values, but we did not capture fluid details. The eAKI rate may be inflated if electrolyte supplementation is included in the eAKI definition. There was cross-site practice variation in prophylactic electrolyte (magnesium and potassium) supplementation. Therefore, the eAKI definition may need to be modified for electrolytes not routinely prophylactically supplemented. Future research should determine how different cisplatin-associated AKI definitions affect associations with long-term outcomes.

Similar to other populations, young age was associated with SCr-AKI.^1,15,38^ Interindividual variability in cisplatin pharmacokinetics and pharmacodynamics and standard practice determine dose of cisplatin based on body size in young children may partly explain this finding.^15,22,39^ Higher baseline estimated GFR was independently associated with AKI. This somewhat paradoxical finding has been consistently described in pediatric AKI studies, without any satisfying explanations.^1,20^ One theory is that children with higher baseline estimated GFR may have lower muscle mass and poor nutrition, although this has never been reported. Another theory is that excessive hydration may dilute SCr; however, this has not been proven, and we did not collect hydration protocol information or mannitol use. In addition, we were unable to assess whether some patients had syndrome of inappropriate antidiuretic hormone secretion, contributing to SCr dilution. Further research should determine the effect of hydration protocols on SCr and cisplatin-associated AKI.

Cisplatin dosage and medications may be associated with AKI. In adults, single-bolus cisplatin infusions are more nephrotoxic than infusions administered for 2 to 5 days.^40,41^ In our study, participants with neuroblastoma received the highest total cisplatin cycle dose, and their protocols contained the highest number of nephrotoxins. These factors could contribute to high AKI risk for neuroblastoma and central nervous system tumors. Synergistic nephrotoxic effects with other drugs remain unclear.^1^ Genetic differences in cisplatin metabolizing enzymes or alterations in cisplatin metabolism from other medications are possible.^42^ In addition, the lower cisplatin infusion dose at the LV may explain the low SCr-AKI rate at LV. We were unable to assess whether there were changes in cisplatin dose, treatment protocol, or fluid administration because of nephrotoxic effects. Furthermore, despite receiving the highest pre-LV cumulative cisplatin dose, no participants with osteosarcoma developed SCr-AKI, likely explaining associations between low prior cumulative cisplatin dose and SCr-AKI at LV. High cumulative dose is typically associated with nephrotoxic effects near cancer treatment end and may be more important in long-term or chronic nephrotoxicity.^8,19,43^ However, the LV infusion dose was higher in participants with SCr-AKI, suggesting that during later individual cisplatin cycles, planned higher dose may be associated with AKI during that cycle. After receiving multiple cisplatin doses, patients may experience unresolved tubular injury, increasing susceptibility of further injury.^44,45^ Although further research is needed with larger sample sizes to differentiate the contributions of risk factors and their associations, identified risk factors should be confirmed for identifying future individual cisplatin-associated AKI risk with a goal of early management. It remains unclear how cisplatin dose should be adjusted because of AKI.

In this pediatric, multicenter study, cisplatin-associated SCr-AKI and eAKI were common and transient; however, long-term clinical and subclinical effects remain unknown. SCr-AKI occurred early during cisplatin treatment, suggesting that AKI monitoring is needed at cisplatin treatment commencement. The identified variables associated with AKI in children receiving cisplatin need validation but represent a potentially applicable AKI risk profile to help clinical decision-making and management (eg, dose, use of other nephrotoxins, and observation duration). Clinical risk profiling may enhance conduct of future cisplatin-associated AKI intervention trials, including antioxidants, kidney blood flow modifiers, and anti-inflammatory mediators.^46,47,48,49,50^

### Strengths and Limitations

This study has strengths, including the prospective, multicenter design, the heterogeneous group of children treated for cancer (enhancing generalizability), and the use of definitions based on standardized AKI definitions with detailed SCr and electrolyte profiles with comprehensive AKI evaluation. This study also has limitations. The modest sample size precluded subgroup analyses (eg, cancer types), limited multivariable analyses, and limited the assessment of confounding and interactions. The study population was predominantly white, limiting race/ethnicity association evaluations. We did not collect detailed information on or evaluate cancer staging and severity or previous cancers and treatments.

## Conclusions

The findings suggest that AKI is common among children receiving cisplatin infusions and that rate and risk factors differ at earlier vs later infusions. These results may help with risk stratification with a goal of risk reduction.

## Notes

Supplement. **eFigure 1.** Diagram of the Study Protocol **eFigure 2.** Relationship Between Age Tertiles and SCr-AKI at EV **eFigure 3.** Serum Phosphorus, Magnesium and Potassium Levels Measured at EV and LV Cisplatin Cycles **eTable 1.** Serum Creatinine (SCr)-Acute Kidney Injury (AKI) Staging Outlined by the Kidney Disease: Improving Global Outcomes (KDIGO) Guidelines and Electrolyte-AKI (eAKI) Grading Based on National Cancer Institute (NCI) Common Terminology Criteria for Adverse Events (CTCAE) Version 4 **eTable 2.** Blood and Urine Collection Success and Analyte Measurements Obtained at the Earlier Cisplatin Visits (EV) and Later Cisplatin Visits (LV) **eTable 3.** Description of Data Collection Success Rate and Missing Data Rate **eTable 4.** Correlation Between Serum Laboratory Values Measured By Routine Care and Study-Specific Values **eTable 5.** Medications given at Baseline, Prior to, 1 Week Before, 3 Days Before and After Cisplatin Infusion at Earlier Cisplatin Visits (EV) and Later Cisplatin Visits (LV) **eTable 6.** Characteristics of Study Population at Earlier Cisplatin Infusion Visit (EV) and Later Cisplatin Infusion Visit (LV), Comparing Patients With Versus Without Electrolyte-Defined AKI (eAKI) With Cisplatin Infusion **eTable 7.** Characteristics of Study Population at Earlier Cisplatin Infusion Visit (EV) and Later Cisplatin Infusion Visit (LV), Comparing Patients With Versus Without AKI Defined by Having Both Serum Creatinine *and* Electrolyte-Defined AKI (SCr + eAKI) With Cisplatin Infusion **eTable 8.** Fractional Excretion of Electrolytes at Earlier Cisplatin Infusion Visit (EV) and Later Cisplatin Infusion Visit (LV) **eTable 9.** Characteristics of Study Participants at EV and LV Stratified by Cancer Type **eTable 10.** Cancer Treatment Details at Earlier Cisplatin Infusion Visit (EV) and Later Cisplatin Infusion Visit (LV) Stratified by Cancer Type
