# Supplementary material for: Epidemiologic Characteristics of Acute Kidney Injury During Cisplatin Infusions in Children Treated for Cancer
Source: JAMA Netw Open. 2020 May 8;3(5):e203639. doi: 10.1001/jamanetworkopen.2020.3639 (PMC7210480; doi:10.1001/jamanetworkopen.2020.3639)
Supplement: Supplement. — eFigure 1. Diagram of the Study Protocol eFigure 2. Relationship Between Age Tertiles and SCr-AKI at EV eFigure 3. Serum Phosphorus, Magnesium and Potassium Levels Measured at EV and LV Cisplatin Cycles eTable 1. Serum Creatinine (SCr)-Acute Kidney Injury (AKI) Staging Outlined by the Kidney Disease: Improving Global Outcomes (KDIGO) Guidelines and Electrolyte-AKI (eAKI) Grading Based on National Cancer Institute (NCI) Common Terminology Criteria for Adverse Events (CTCAE) Version 4 eTable 2. Blood and Urine Collection Success and Analyte Measurements Obtained at the Earlier Cisplatin Visits (EV) and Later Cisplatin Visits (LV) eTable 3. Description of Data Collection Success Rate and Missing Data Rate eTable 4. Correlation Between Serum Laboratory Values Measured By Routine Care and Study-Specific Values eTable 5. Medications given at Baseline, Prior to, 1 Week Before, 3 Days Before and After Cisplatin Infusion at Earlier Cisplatin Visits (EV) and Later Cisplatin Visits (LV) eTable 6. Characteristics of Study Population at Earlier Cisplatin Infusion Visit (EV) and Later Cisplatin Infusion Visit (LV), Comparing Patients With Versus Without Electrolyte-Defined AKI (eAKI) With Cisplatin Infusion eTable 7. Characteristics of Study Population at Earlier Cisplatin Infusion Visit (EV) and Later Cisplatin Infusion Visit (LV), Comparing Patients With Versus Without AKI Defined by Having Both Serum Creatinine and Electrolyte-Defined AKI (SCr + eAKI) With Cisplatin Infusion eTable 8. Fractional Excretion of Electrolytes at Earlier Cisplatin Infusion Visit (EV) and Later Cisplatin Infusion Visit (LV) eTable 9. Characteristics of Study Participants at EV and LV Stratified by Cancer Type eTable 10. Cancer Treatment Details at Earlier Cisplatin Infusion Visit (EV) and Later Cisplatin Infusion Visit (LV) Stratified by Cancer Type [file jamanetwopen-3-e203639-s001.pdf]

Supplementary Online Content

McMahon KR, Rassekh SR, Schultz KR, et al; Applying Biomarkers to Minimize Long-term Effects of Childhood/Adolescent Cancer Treatment (ABLE) Research Study Group. Epidemiologic characteristics of acute kidney injury during cisplatin infusions in children treated for cancer. *JAMA Netw Open*. 2020;3(5):e203639. doi:10.1001/jamanetworkopen.2020.3639

eFigure 1. Diagram of the Study Protocol

eFigure 2. Relationship Between Age Tertiles and SCr-AKI at EV

eFigure 3. Serum Phosphorus, Magnesium and Potassium Levels Measured at the EV and the LV Cisplatin Cycles

eTable 1. Serum Creatinine (SCr)-Acute Kidney Injury (AKI) Staging Outlined by the Kidney Disease: Improving Global Outcomes (KDIGO) Guidelines and Electrolyte-AKI (eAKI) Grading Based on National Cancer Institute (NCI) Common Terminology Criteria for Adverse Events (CTCAE) Version 4[21,31]

eTable 2. Blood and Urine Collection Success and Analyte Measurements Obtained at the Earlier Cisplatin Visits (EV) and Later Cisplatin Visits (LV)

eTable 3. Description of Data Collection Success Rate and Missing Data Rate

eTable 4. Correlation Between Serum Laboratory Values Measured By Routine Care and Study-Specific Values

eTable 5. Medications given at Baseline, Prior to, 1 Week Before, 3 Days Before and After Cisplatin Infusion at Earlier Cisplatin Visits (EV) and Later Cisplatin Visits (LV)

eTable 6. Characteristics of Study Population at Earlier Cisplatin Infusion Visit (EV) and Later Cisplatin Infusion Visit (LV), Comparing Patients With Versus Without Electrolyte-Defined AKI (eAKI) With Cisplatin Infusion

eTable 7. Characteristics of Study Population at Earlier Cisplatin Infusion Visit (EV) and Later Cisplatin Infusion Visit (LV), Comparing Patients With Versus Without AKI Defined by Having Both Serum Creatinine *and* Electrolyte-Defined AKI (SCr plus eAKI) With Cisplatin Infusion

eTable 8. Fractional Excretion of Electrolytes at Earlier Cisplatin Infusion Visit (EV) and Later Cisplatin Infusion Visit (LV)

eTable 9. Characteristics of Study Participants at EV and LV Stratified by Cancer Type

eTable 10. Cancer Treatment Details at Earlier Cisplatin Infusion Visit (EV) and Later Cisplatin Infusion Visit (LV) Stratified by Cancer Type

This supplementary material has been provided by the authors to give readers additional information about their work.

© 2020 McMahon KR et al. JAMA Network Open.

eFigure 1. Diagram of the Study Protocol.

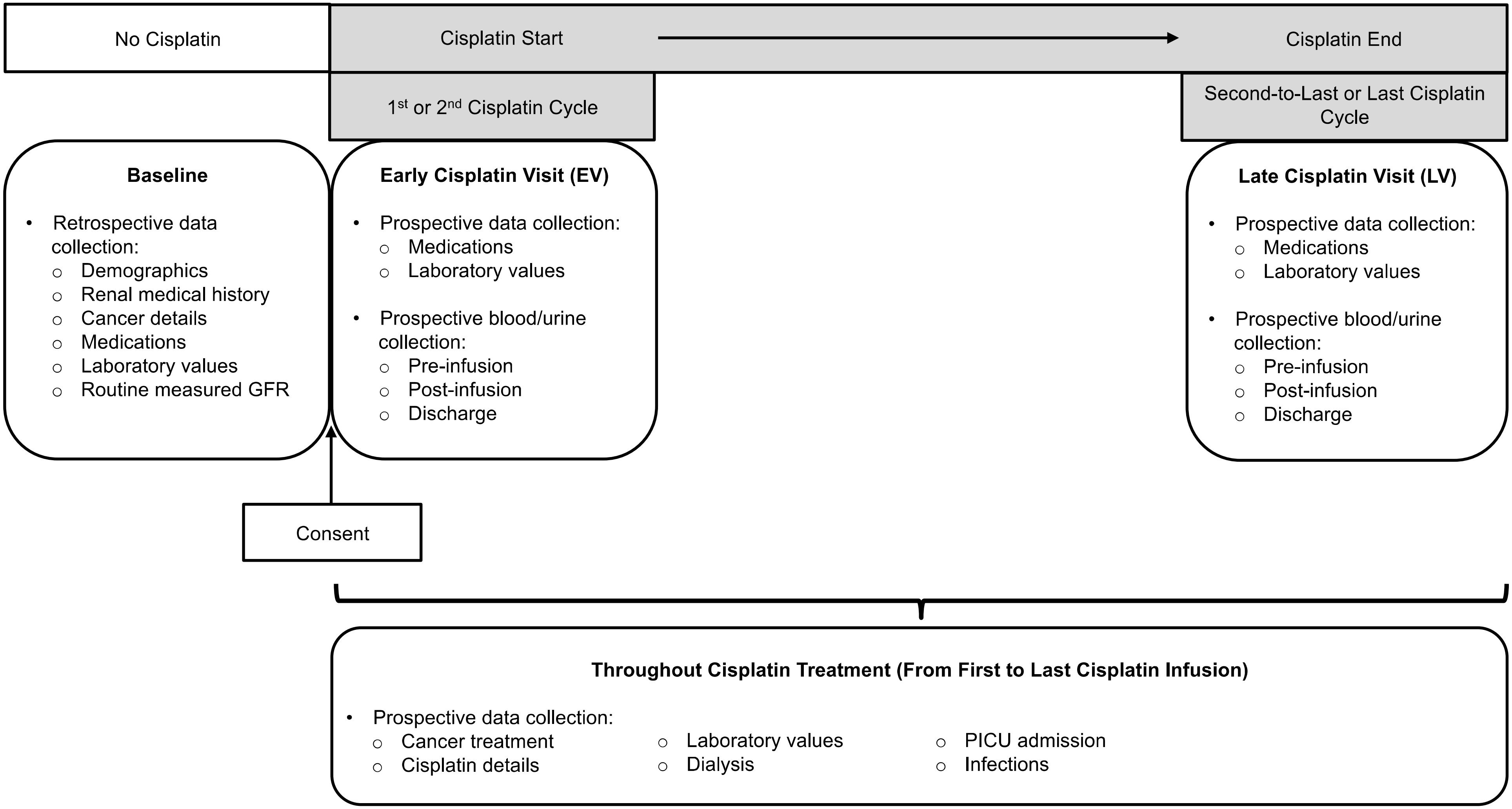

eFigure 1 Legend: Time points for specimen and data collection in reference to cisplatin treatment. Data was collected from baseline to end of cisplatin treatment, including between Early and Late Cisplatin Visits (between EV and LV).

Abbreviations: *GFR*: glomerular filtration rate; *PICU*: pediatric intensive care unit.

© 2020 McMahon KR et al. JAMA Network Open.

eFigure 2. Relationship between Age Tertiles and SCr-AKI at EV.

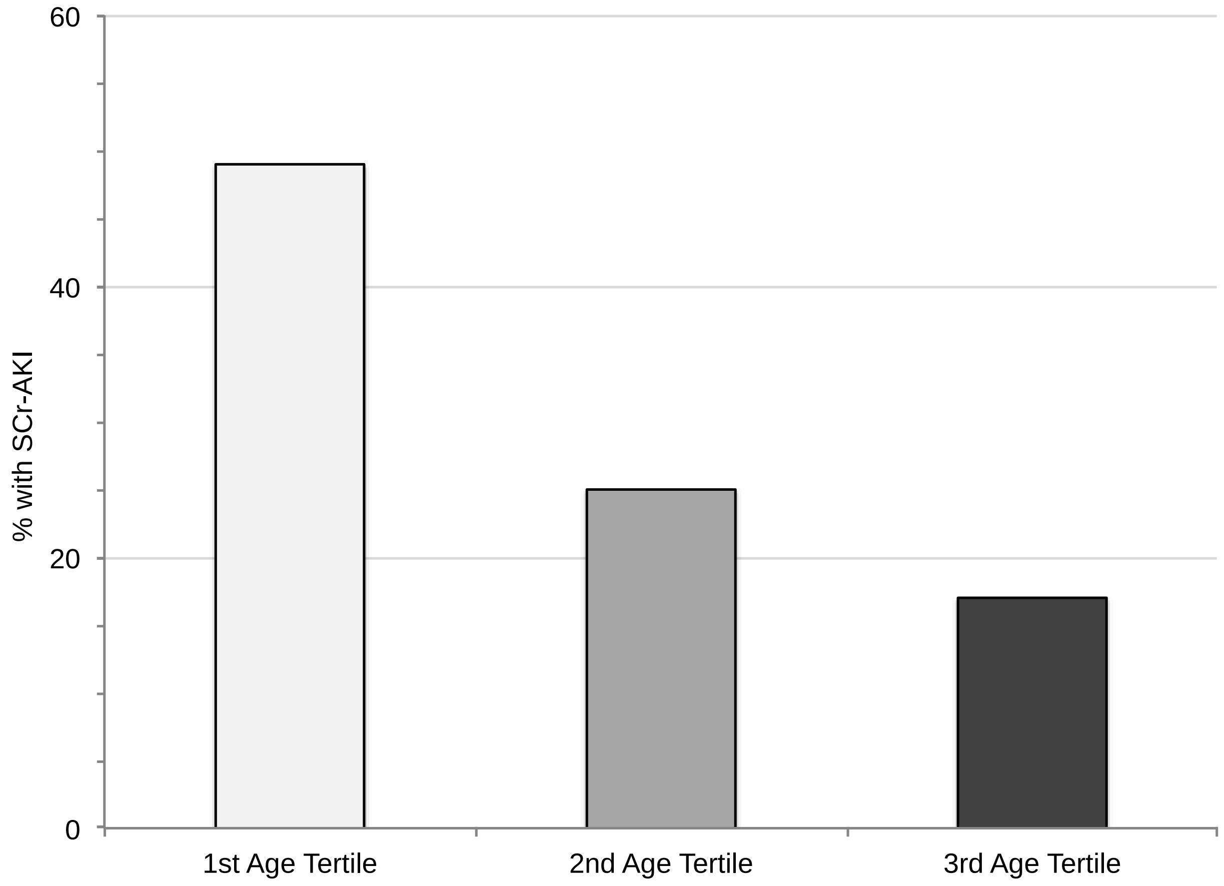

eFigure 2 Legend: The proportion of study participants with SCr-AKI at EV increases with decreasing age tertile. Chi-square p-value was .001. 1st Age Tertile (age ≤ 3 years): 26/53 (49%) study participants with SCr-AKI. 2nd Age Tertile (age > 3 and ≤ 9.2 years): 13/53 (25%) participants with SCr-AKI. 3rd Age Tertile (age > 9.2 years): 9/53 (17%) participants with SCr-AKI.

Abbreviations: *SCr*: Serum Creatinine; *AKI*: Acute Kidney Injury.

© 2020 McMahon KR et al. JAMA Network Open.

eFigure 3. Serum Phosphorus, Magnesium and Potassium Levels Measured at the EV and the LV Cisplatin Cycles.

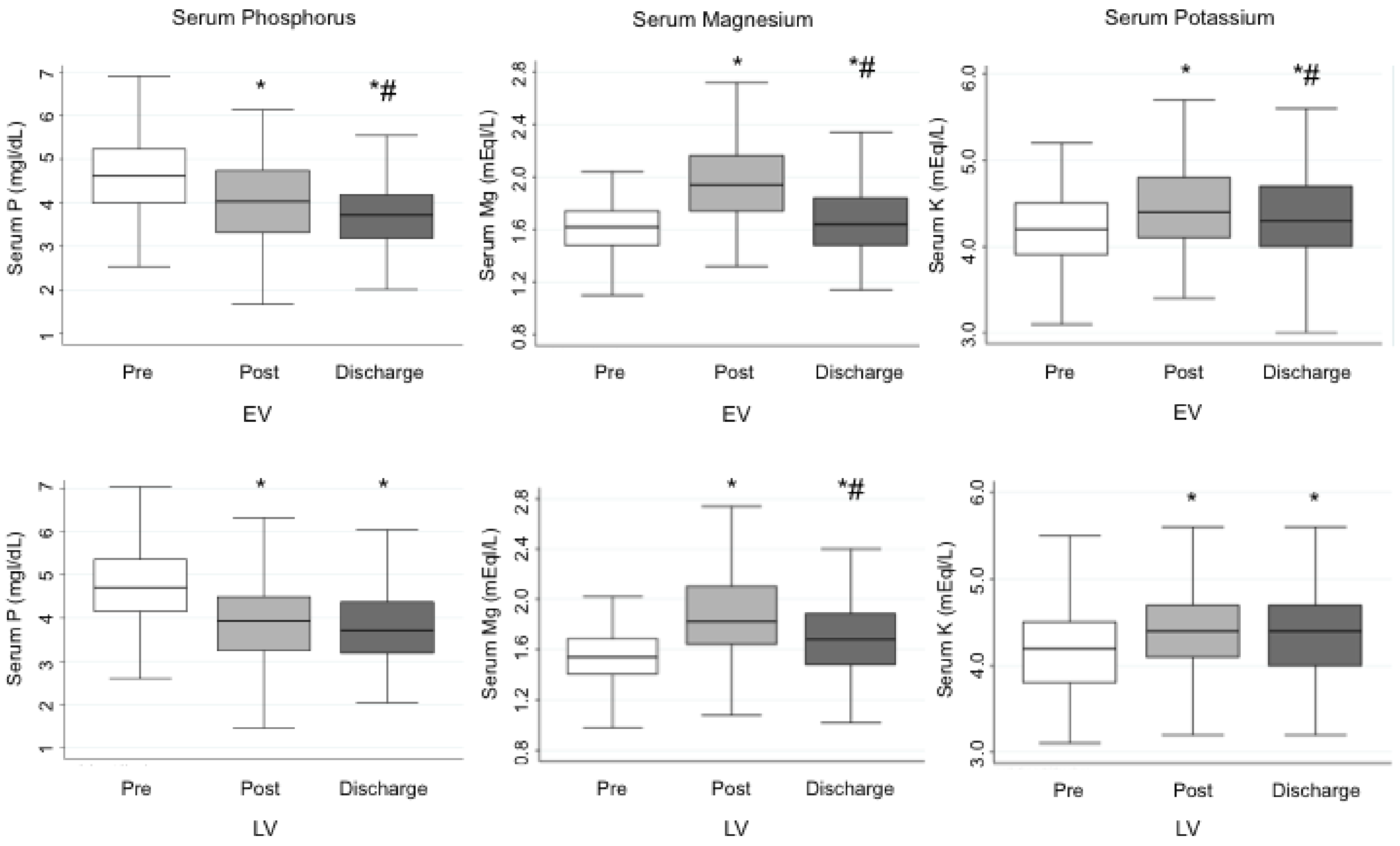

© 2020 McMahon KR et al. JAMA Network Open.

eFigure 3 Legend: Box-and-whisker plots (the lower end of the boxes represents the 25th percentile; the upper end of the boxes represents the 75th percentile; the middle line is the median; the upper whisker outlines the upper adjacent value; the lower whisker outlines the lower adjacent value) of serum electrolytes (magnesium, potassium and phosphorus) measured at three time points surrounding the cisplatin infusion at EV (top row) and LV (bottom row). Pre (pre-infusion): serum collected on the day of the infusion before infusion start. Post (post-infusion): serum collected the morning after the cisplatin infusion. Discharge: serum collected just before discharge from hospital (study day 2 to 5). EV Pre: n=157; EV Post: n=155; EV Discharge: n=151. LV Pre: n=127; LV Post: n=127; LV Discharge: n=122. To convert magnesium, phosphorus and potassium from conventional units to *Système International* (SI) units, conversion factors are 0.5, 0.323 and 1, respectively. Multiply conventional units by conversion factor to obtain SI units (mmol/L).

*Indicates a significant difference from the Pre time point by Wilcoxon signed-rank test ($p<.05$).

#Indicates a significant difference from the Post time point by Wilcoxon signed-rank test ($p<.05$).

Abbreviations: *EV*: Early Cisplatin Visit; *LV*: Late Cisplatin Visit; *P*: Phosphorus; *Mg*: Magnesium; *K*: Potassium.

© 2020 McMahon KR et al. JAMA Network Open.

eTable 1. Serum Creatinine (SCr)-Acute Kidney Injury (AKI) Staging outlined by the Kidney Disease: Improving Global Outcomes (KDIGO) Guidelines and Electrolyte-AKI (eAKI) Grading based on National Cancer Institute (NCI) Common Terminology Criteria for Adverse Events (CTCAE) Version 4[21,31].

<table>
<tr><td colspan="4">SCr-AKI Staging (KDIGO Definition)</td></tr>
<tr><td>Stage</td><td colspan="3">SCr</td></tr>
<tr><td>0</td><td colspan="3"><1.5 times baseline</td></tr>
<tr><td rowspan="2">1</td><td colspan="3">1.5-1.9 times baseline OR</td></tr>
<tr><td colspan="3">≥0.3 mg/dl (≥26.5 μmol/l) increase</td></tr>
<tr><td>2</td><td colspan="3">2.0-2.9 times baseline</td></tr>
<tr><td rowspan="4">3</td><td colspan="3">3.0 times baseline OR</td></tr>
<tr><td colspan="3">Increase in SCr to ≥4.0 mg/dl (≥353.6 μmol/l) OR</td></tr>
<tr><td colspan="3">Initiation of renal replacement therapy OR</td></tr>
<tr><td colspan="3">In patients <18 years, decrease in eGFR to <35 mL/min/1.73m^2</td></tr>
<tr><td colspan="4">Electrolyte-AKI Grading (NCI CTCAE)</td></tr>
<tr><td>Grade</td><td>Magnesium, mEq/L</td><td>Phosphorus, mg/dL</td><td>Potassium, mEq/L</td></tr>
<tr><td rowspan="6">0</td><td>≥LLN[a]:</td><td>≥LLN[a]:</td><td>≥LLN[a]:</td></tr>
<tr><td>Age ≥0 to <0.0192 years: ≥1.2</td><td>Age ≥0 to <1 year: ≥4.7</td><td>Age ≥0 to <0.083 years: ≥3.9</td></tr>
<tr><td rowspan="3">Age ≥0.0192 to <18 years: ≥1.4</td><td>Age ≥1 to <5 years: ≥3.4</td><td>Age ≥0.083 to <1 year: ≥3.6</td></tr>
<tr><td>Age ≥5 to <10 years: ≥2.9</td><td>Age ≥1 to <5 years: ≥3.2</td></tr>
<tr><td>Age ≥10 to <18 years: ≥3.3</td><td>Age ≥5 to <10 years: ≥3.4</td></tr>
<tr><td>Age ≥18 years: ≥1.5</td><td>Age ≥18 years: ≥2.9</td><td>Age ≥10 years: ≥3.5</td></tr>
<tr><td rowspan="6">1</td><td><LLN – 1.0[a]:</td><td><LLN - 2.5[a]:</td><td><LLN - 3.0[a]:</td></tr>
<tr><td>Age ≥0 to <0.0192 years: <1.2 - 1.0</td><td>Age ≥0 to <1 year: <4.7 - 2.5</td><td>Age ≥0 to <0.083 years: <3.9 - 3.0</td></tr>
<tr><td rowspan="3">Age ≥0.0192 to <18 years: <1.4 - 1.0</td><td>Age ≥1 to <5 years: <3.4 - 2.5</td><td>Age ≥0.083 to <1 year: <3.6 - 3.0</td></tr>
<tr><td>Age ≥5 to <10 years: <2.9 - 2.5</td><td>Age ≥1 to <5 years: <3.2 - 3.0</td></tr>
<tr><td>Age ≥10 to <18 years: <3.3 - 2.5</td><td>Age ≥5 to <10 years: <3.4 - 3.0</td></tr>
<tr><td>Age ≥18 years: <1.5 - 1.0</td><td>Age ≥18 years: <2.9 - 2.5</td><td>Age ≥10 years: <3.5 - 3.0</td></tr>
<tr><td>2</td><td><1.0 - 0.8</td><td><2.5 - 2.0</td><td>---</td></tr>
<tr><td>3</td><td><0.8 - 0.6</td><td><2.0 - 1.0</td><td><3.0 - 2.5</td></tr>
<tr><td>4</td><td><0.6</td><td><1.0</td><td><2.5</td></tr>
</table>

© 2020 McMahon KR et al. JAMA Network Open.

To convert magnesium, phosphorus and potassium from conventional units to *Système International* (SI) units, conversion factors are 0.5, 0.323 and 1, respectively. Multiply conventional units by conversion factor to obtain SI units (mmol/L).

[a]Laboratory specific values for McGill University Health Centre were used to define LLN for age (shown in table).

Abbreviations: *SCr:* Serum creatinine; *KDIGO*: Kidney Disease: Improving Global Outcomes; *AKI*: Acute kidney injury; *eGFR*: Estimated glomerular filtration rate; *NCI*: National Cancer Institute; *LLN*: Lower limit of normal for age.

© 2020 McMahon KR et al. JAMA Network Open.

eTable 2. Blood and Urine Collection Success and Analyte Measurements Obtained at the Earlier Cisplatin Visits (EV) and Later Cisplatin Visits (LV).

Specimen Collection[a, b]						
	**EV (n=159)**			**LV (n=143)**		
	**Pre**	**Post**	**Discharge**	**Pre**	**Post**	**Discharge**
Blood *and* Urine	153 (96)	152 (96)	146 (92)	122 (85)	123 (86)	119 (83)
Blood *or* Urine	158 (99)	156 (98)	153 (96)	127 (89)	128 (90)	123 (86)
Blood only	157 (99)	155 (97)	151 (95)	127 (89)	127 (89)	123 (86)
Urine only	154 (97)	153 (96)	148 (93)	122 (85)	124 (87)	119 (83)
Analyte Measurements[a, c, d]						
	**EV (n=159)**			**LV (n=143)**		
	**Pre**	**Post**	**Discharge**	**Pre**	**Post**	**Discharge**
Blood *and* Urine	149 (94)[e]	147 (92)	144 (91)	120 (84)	122 (85)	116 (81)[f]
Blood *or* Urine	158 (99)[e]	156 (98)	153 (96)	127 (89)	128 (90)	123 (86)[f]
Blood only	157 (99)	155 (97)	151 (95)	127 (89)	127 (89)	123 (86)[f]
Urine only	150 (94)[e]	148 (93)	146 (92)	120 (84)	123 (86)	116 (81)

[a]Results are expressed as No. (%) for each study sampling time point.

[b] Reasons for specimen non-collection included insufficient quantity, participant refusal, sample misplaced and protocol error.

[c]Analyte measurements refer to creatinine, magnesium, potassium and phosphorous.

[d]Missing analyte measurements are due to insufficient quantity of specimen available.

[e]One study participant did not have enough urine to measure urinary phosphorous for EV pre-infusion.

[f]For one study participant at LV discharge, no serum electrolytes (phosphorous, potassium, magnesium) results were reported by the laboratory due to severe hemolysis.

Abbreviations: *EV*: Early Cisplatin Visit; *LV*: Late Cisplatin Visit; *Pre*: Pre-infusion; *Post*: Post-infusion; *Discharge*: hospital discharge.

© 2020 McMahon KR et al. JAMA Network Open.

eTable 3. Description of Data Collection Success Rate and Missing Data Rate.

Data Collection				
	**EV (n=159)**		**LV (n=143)**	
	**Data Collected, No. (%)[a]**	**Data Not Documented or Unknown, No. (%)[a]**	**Data Collected, No. (%)[a]**	**Data Not Documented or Unknown, No. (%)[a]**
Participant Characteristics and Potential AKI Risk Factors Data				
**Baseline (prior to 1st cisplatin infusion)**				
Sex	159 (100)	0 (0)		
Race	159 (100)	0 (0)		
Cancer Diagnosis	159 (100)	0 (0)		
Cancer involves one or both kidneys	159 (100)	2 (1)		
Renal medical history	159 (100)	0 (0)		
Hypertension	159 (100)	2 (1)		
CKD	159 (100)	6 (4)		
Dialysis	159 (100)	2 (1)		
Congenital renal anomaly	159 (100)	3 (2)		
Vesicoureteral reflux/ Urinary tract infection	159 (100)	4 (3)		
Other Renal Medical History[b]	159 (100)	1 (1)		
Nephrotoxic drugs prior to 1st cisplatin[c]	159 (100)	0 (0)		
Acyclovir	159 (100)	9 (6)		
Amphotericin	159 (100)	10 (6)		
Aminoglycosides	159 (100)	10 (6)		
Vancomycin	159 (100)	10 (6)		
ACE inhibitor	159 (100)	10 (6)		
Ganciclovir/Valganciclovir	159 (100)	10 (6)		
Ifosfamide	159 (100)	5 (3)		
Methotrexate	159 (100)	8 (5)		
Loop diuretics[d]	159 (100)	9 (6)		
NSAIDs or ASA[e]	159 (100)	22 (14)		
**Immediately Prior to/Day of EV/LV Infusion**				
Age at EV/LV	159 (100)	0 (0)	143 (100)	0 (0)
Cisplatin naïve at EV	159 (100)	0 (0)		
Pre-visit eGFR	159 (100)	0 (0)	143 (100)	0 (0)
Pre-infusion serum phosphorus[f]	159 (100)	0 (0)	141 (99)	2 (1)
Pre-infusion serum magnesium[f]	159 (100)	0 (0)	141 (99)	2 (1)
Pre-infusion serum potassium[f]	159 (100)	0 (0)	143 (100)	0 (0)

© 2020 McMahon KR et al. JAMA Network Open.

	EV (n=159)		LV (n=143)	
	Data Collected, No. (%)[a]	Data Not Documented or Unknown, No. (%)[a]	Data Collected, No. (%)[a]	Data Not Documented or Unknown, No. (%)[a]
**Immediately Prior to/Day of EV/LV Infusion (continued)**				
LV was the last cisplatin cycle of cancer treatment			143 (100)	0 (0)
Cumulative cisplatin dose prior to EV/LV	159 (100)	0 (0)	143 (100)	0 (0)
EV/LV cisplatin infusion dose	159 (100)	0 (0)	143 (100)	0 (0)
Electrolyte supplements in 3 days prior to EV/LV[g]	159 (100)	0 (0)	143 (100)	0 (0)
Infection prior to EV/LV	159 (100)	0 (0)	143 (100)	0 (0)
PICU Admission prior to EV/LV	159 (100)	0 (0)	143 (100)	0 (0)
SCr-AKI episode prior to EV/LV	159 (100)	46 (29)	143 (100)	0 (0)
eAKI episode prior to EV/LV	159 (100)	0 (0)	143 (100)	0 (0)
Days between EV and LV visits			143 (100)	0 (0)
Concurrent nephrotoxins at EV/LV[h]	159 (100)	0 (0)	143 (100)	0 (0)
**Cancer Treatment Details**				
EV/LV total cisplatin cycle dose	159 (100)	0 (0)	143 (100)	0 (0)
Any flank (left or right), whole abdomen, pelvic or total body radiation planned	159 (100)	1 (1)	143 (100)	1 (1)
**Post-Cisplatin**				
EV/LV length of stay	159 (100)	0 (0)	143 (100)	0 (0)
Nephrotoxins in 10 days post EV/LV[i]	159 (100)	0 (0)	143 (100)	0 (0)
Electrolyte supplements in 10 days post EV/LV[g]	159 (100)	0 (0)	143 (100)	0 (0)
SCr-AKI Data				
Baseline SCr	159 (100)	0 (0)	143 (100)	0 (0)
Peak SCr (in 10 days post-infusion)	159 (100)	0 (0)	143 (100)	0 (0)
SCr-AKI Status	159 (100)	0 (0)	143 (100)	0 (0)
eAKI Data				
Nadir Serum Phosphorous (in 10 days post-infusion)	158 (99)	1 (1)	143 (100)	0 (0)
Nadir Serum Magnesium (in 10 days post-infusion)	158 (99)	1 (1)	143 (100)	0 (0)
Nadir Serum Potassium (in 10 days post-infusion)	159 (100)	0 (0)	143 (100)	0 (0)
eAKI Status	159 (100)	0 (0)	143 (100)	0 (0)

Shaded cells are not applicable for that time point.

© 2020 McMahon KR et al. JAMA Network Open.

[a]Results are expressed as No. (%) for each study sampling time point; percentages are based on the total for each column

[b]Including family history of kidney disease, kidney stones, serum electrolyte abnormality requiring treatment or AKI.

[c]Receipt of acyclovir, amphotericin, aminoglycosides (gentamycin, tobramycin, amikacin), vancomycin, angiotensin converting enzyme inhibitor, ganciclovir/valganciclovir, ifosfamide or methotrexate in 2 weeks pre-cisplatin commencement.

[d]Furosemide, ethacrinic acid, bumetanide.

[e]Ibuprofen, naproxen, cox-inhibitors, diclofenac, ketorolac or any form of aspirin.

[f]Pre-infusion electrolyte was determined by using the study-measured electrolyte (measured on day of cisplatin infusion, pre-infusion); if unavailable, the most recent available routine electrolyte value was used (if the study-measured electrolyte was unavailable, the routine electrolyte value from the day of cisplatin infusion, pre-infusion was used; if unavailable, the routine electrolyte value from the day before, 2 days before and 3 days before cisplatin infusion was used, respectively

[g]Any form of oral or IV supplementation of magnesium, potassium or phosphorus (excluding total parenteral nutrition).

[h]Acyclovir, amphotericin, aminoglycosides (gentamycin, tobramycin, amikacin), ifosfamide, or chemotherapy protocol indicates a nephrotoxin (aldesleukin, busulfan, carboplatin, dinutuximab, gemcitabine, ifosfamide, lomustine, melphalan, methotrexate, radiotherapy, rituximab, stem cell transplant or temsirolimus) was given within 24h of cisplatin infusion.

[i]Acyclovir, amphotericin, aminoglycosides (gentamycin, tobramycin, amikacin), ifosfamide

Abbreviations: *EV*: Early Cisplatin Visit; *LV*: Late Cisplatin Visit; *CKD*: Chronic Kidney Disease; *ACE*: Angiotensin converting enzyme; *NSAIDs*: Nonsteroidal anti-inflammatory drugs; *ASA*: Acetylsalicylic acid; *eGFR: Estimated glomerular filtration rate by equation; PICU: Pediatric intensive care unit; SCr: Serum Creatinine; AKI: Acute Kidney Injury; eAKI: Electrolyte-AKI.*

© 2020 McMahon KR et al. JAMA Network Open.

eTable 4. Correlation Between Serum Laboratory Values Measured By Routine Care and Study-Specific Values.

EV[a]				
	**Spearman rho**	**No. of patients**	**Routine, Median [IQR]**	**Study-Measured, Median [IQR]**
Pre P	0.61	104	4.8 [4.3-5.4] mg/dL	4.7 [4.1-5.2] mg/dL
Post P	0.77	122	4.0 [3.4-4.6] mg/dL	4.1 [3.4-4.8] mg/dL
Discharge P	0.73	115	3.7 [3.3-4.3] mg/dL	3.8 [3.2-4.2] mg/dL
Pre Mg	0.68	111	1.6 [1.5-1.7] mEq/L	1.6 [1.5-1.7] mEq/L
Post Mg	0.75	128	1.9 [1.7-2.1] mEq/L	1.9 [1.7-2.2] mEq/L
Discharge Mg	0.70	127	1.7 [1.5-1.8] mEq/L	1.6 [1.5-1.8] mEq/L
Pre K	0.40	118	4.1 [3.9-4.3] mEq/L	4.2 [4.0-4.5] mEq/L
Post K	0.58	137	4.1 [3.9-4.4] mEq/L	4.5 [4.2-4.8] mEq/L
Discharge K	0.64	134	4.2 [3.8-4.5] mEq/L	4.3 [4.0-4.7] mEq/L
Pre SCr	0.86	122	0.4 [0.3-0.5] mg/dL	0.3 [0.2-0.5] mg/dL
Post SCr	0.88	133	0.3 [0.2-0.4] mg/dL	0.3 [0.2-0.4] mg/dL
Discharge SCr	0.91	132	0.3 [0.2-0.5] mg/dL	0.3 [0.3-.0.5] mg/dL
**LV[a]**				
Pre P	0.70	87	5.0 [4.3-5.8] mg/dL	4.7 [4.1-5.3] mg/dL
Post P	0.75	96	4.1 [3.6-4.5] mg/dL	4.0 [3.3-4.5] mg/dL
Discharge P	0.77	92	4.0 [3.3-4.4] mg/dL	3.7 [3.1-4.4] mg/dL
Pre Mg	0.55	90	1.5 [1.4-1.6] mEq/L	1.5 [1.4-1.7] mEq/L
Post Mg	0.79	104	1.8 [1.6-2.0] mEq/L	1.8 [1.7-2.1] mEq/L
Discharge Mg	0.76	96	1.6 [1.4-1.8] mEq/L	1.6 [1.5-1.9] mEq/L
Pre K	0.33	95	4.1 [3.9-4.3] mEq/L	4.2 [3.8-4.5] mEq/L
Post K	0.47	113	4.2 [3.9-4.4] mEq/L	4.4 [4.2-4.7] mEq/L
Discharge K	0.41	105	4.2 [4.0-4.4] mEq/L	4.4 [4.1-4.7] mEq/L
Pre SCr	0.88	95	0.4 [0.3-0.5] mg/dL	0.3 [0.2-0.5] mg/dL
Post SCr	0.91	111	0.3 [0.2-0.5] mg/dL	0.3 [0.2-0.4] mg/dL
Discharge SCr	0.89	106	0.3 [0.2-0.5] mg/dL	0.3 [0.2-0.4] mg/dL

© 2020 McMahon KR et al. JAMA Network Open.

To convert creatinine, magnesium, phosphorus and potassium from conventional units to *Système International* (SI) units, conversion factors are 88.4, 0.5, 0.323 and 1, respectively. Multiply conventional units by conversion factor to obtain SI units (mmol/L for electrolytes; µmol/L for creatinine).

[a] Correlations for each time point were done using routine and study-measured values that occurred on the same day only.

Abbreviations: *EV*: Early Cisplatin Visit; *LV*: Late Cisplatin Visit; *P*: Phosphorus; *Mg*: Magnesium; *K*: Potassium; *SCr*: Serum creatinine; *Pre*: Pre-infusion; *Post*: Post-infusion; *Discharge*: hospital discharge.

© 2020 McMahon KR et al. JAMA Network Open.

eTable 5. Medications given at Baseline, Prior to, 1 Week Before, 3 Days Before and After Cisplatin Infusion at Earlier Cisplatin Visits (EV) and Later Cisplatin Visits (LV).

**EV**				
**Baseline (In 2 weeks before 1st cisplatin infusion)**				
	**Patients, No. (%)[a]**			
**Drug**	**All (n=159)**	**EV SCr-AKI (n=48)**	**No EV SCr-AKI (n=111)**	***P* value**
Acyclovir	2 (1)	0 (0)	2 (2)	1.00
Amphotericin	1 (1)	1 (2)	0 (0)	.30
Aminoglycosides	7 (4)	4 (8)	3 (3)	.20
Vancomycin	8 (5)	5 (10)	3 (3)	.06
ACE inhibitor	0 (0)	0 (0)	0 (0)	-
Ganciclovir/Valganciclovir	0 (0)	0 (0)	0 (0)	-
Loop diuretics[b]	5 (3)	4 (8)[c]	1 (1)	.03
NSAIDs or ASA[d]	18 (11)	3 (6)	15 (14)	.28
Ifosfamide	0 (0)	0 (0)	0 (0)	-
Methotrexate	11 (7)	4 (8)	7 (6)	.74
**Prior to EV**				
Nephrotoxin in chemotherapy protocol[e]	67 (42)	17 (35)	50 (45)	.26
**1 Week Before EV**				
Nephrotoxin in chemotherapy protocol[e]	29 (18)	6 (13)	23 (21)	.22
**3 Days Before EV Cisplatin Infusion**				
Acyclovir	2 (1)	0 (0)	2 (2)	1.00
Amphotericin	0 (0)	0 (0)	0 (0)	-
Aminoglycosides	2 (1)	1 (2)	1 (1)	.51
Ifosfamide	0 (0)	0 (0)	0 (0)	-
Loop diuretics[b]	4 (3)	3 (6)	1 (1)	.08
NSAID or ASA[d]	5 (3)	1 (2)	4 (4)	1.00
**1 Week Post-EV Cisplatin Infusion**				
Nephrotoxin in chemotherapy protocol[e]	26 (16)	6 (13)	20 (18)	.39
**10 Days Post-EV Cisplatin Infusion**				
Acyclovir	3 (2)	0 (0)	3 (3)	.55
Amphotericin	4 (3)	3 (6)	1 (1)	.08
Aminoglycosides	8 (5)	4 (8)	4 (4)	.24
Ifosfamide	0 (0)	0 (0)	0 (0)	-
Loop diuretics[b]	14 (9)	5 (10)	9 (8)	.64
NSAIDs or ASA[d]	1 (1)	1 (2)	0 (0)	.30

© 2020 McMahon KR et al. JAMA Network Open.

LV				
**Drug**	**ALL (n=143)**	**LV SCr-AKI (n=23)**	**No LV SCr-AKI (n=120)**	***P* value**
**Prior to LV**				
Nephrotoxin in chemotherapy protocol[e]	79 (55)	9 (39)	70 (58)	.09
**1 Week Before LV**				
Nephrotoxin in chemotherapy protocol[e]	31 (22)	3 (13)	28 (23)	.41
**3 Days Before LV Cisplatin Infusion**				
Acyclovir	3 (2)	1 (4)	2 (2)	.41
Amphotericin	0 (0)	0 (0)	0 (0)	-
Aminoglycosides	1 (1)	0 (0)	1 (1)	1.00
Ifosfamide	0 (0)	0 (0)	0 (0)	-
Loop diuretics[b]	1 (1)	0 (0)	1 (1)	1.00
NSAID or ASA[d]	2 (1)	0 (0)	2 (2)	1.00
**1 Week Post-LV Cisplatin Infusion**				
Nephrotoxin in chemotherapy protocol[e]	25 (17)	3 (13)	22 (18)	.77
**10 Days Post-LV Cisplatin Infusion**				
Acyclovir	3 (2)	1 (4)	2 (2)	.41
Amphotericin	1 (1)	0 (0)	1 (1)	1.00
Aminoglycosides	8 (6)	1 (4)	7 (6)	1.00
Ifosfamide	0 (0)	0 (0)	0 (0)	-
Loop diuretics[b]	16 (11)	3 (13)	13 (11)	.72
NSAIDs or ASA[d]	1 (1)	0 (0)	1 (1)	1.00

[a]Percentages are based on the total for each column.

[b]Furosemide, ethacrinic acid, bumetanide.

[c]Indicates significant difference between SCr-AKI and Non-AKI groups for that time point ($p<.05$).

[d]Ibuprofen, naproxen, cox-inhibitors, diclofenac, ketorolac or any form of aspirin.

[e]Chemotherapy protocol was considered to contain other nephrotoxins if any of the following were included in protocol: aldesleukin, busulfan, carboplatin, dinutuximab, gemcitabine, ifosfamide, lomustine, melphalan, methotrexate, radiotherapy, rituximab, stem cell transplant or temsirolimus.

Abbreviations: *EV*: Early Cisplatin Visit; *LV*: Late Cisplatin Visit; *SCr*: Serum Creatinine; *AKI*: Acute Kidney Injury; *ACE*: angiotensin converting enzyme; *NSAIDs*: Nonsteroidal anti-inflammatory drugs; *ASA*: acetylsalicylic acid.

© 2020 McMahon KR et al. JAMA Network Open.

eTable 6. Characteristics of Study Population at Earlier Cisplatin Infusion Visit (EV) and Later Cisplatin Infusion Visit (LV), Comparing Patients With Versus Without Electrolyte-Defined AKI (eAKI) With Cisplatin Infusion.

	EV		LV	
**Characteristic**	**eAKI (n=106)[a]**	**No eAKI (n=53)[a]**	**eAKI (n=100)[a]**	**No eAKI (n=43)[a]**
**Baseline (prior to 1st cisplatin infusion)**				
Male, No. (%)	47 (44)[b]	33 (62)	56 (56)[b]	13 (30)
White Race, No. (%)	81 (76)	36 (68)	76 (76)	31 (72)
Cancer diagnosis, No. (%)				
CNS Tumor[c]	41 (39)	17 (32)	39 (39)	16 (37)
Neuroblastoma	22 (21)	21 (40)	18 (18)	13 (30)
Osteosarcoma	25 (24)	8 (15)	27 (27)	6 (14)
Germ cell tumor	11 (10)	3 (6)	10 (10)	4 (9)
Hepatoblastoma	6 (6)	3 (6)	5 (5)	4 (9)
Other[d]	1 (1)	1 (2)	1 (1)	0 (0)
Cancer involves one or both kidneys, No. (%)	6 (6)	5 (9)	3 (3)	4 (9)
Renal medical history, No. (%)[e]	10 (9)	3 (6)	5 (5)	6 (14)
Any nephrotoxic drug prior to 1st cisplatin, No. (%)[f]	18 (17)	9 (17)	N/A	N/A
Vancomycin in 2 weeks pre-cisplatin, No. (%)	6 (6)	2 (4)	N/A	N/A
**Immediately Prior to/Day of EV/LV Infusion**				
Age at EV/LV, median [IQR], years	6.3 [2.4-13.0]	4.1 [2.0-8.9]	8.6 [2.7-13.7][b]	4.1 [2.2-6.6]
Age at EV/LV <3 years, No. (%)	31 (29)	21 (40)	27 (27)	18 (42)
Cisplatin naïve at EV, No. (%)	58 (55)	33 (62)	N/A	N/A
Pre-visit eGFR, median [IQR], mL/min/1.73m^2 [g]	139 [118-171]	143 [128-164]	133 [113-165][b]	149 [129-182]
Pre-infusion serum phosphorus, mean (SD), mg/dL[h]	4.5 (1.0)[b]	5.0 (1.4)	4.7 (0.9)[i]	4.9 (0.8)
Pre-infusion serum magnesium, mean (SD), mEq/L[h]	1.6 (0.2)	1.6 (0.2)	1.5 (0.2)	1.6 (0.2)[j]
LV was the last cisplatin cycle of cancer treatment, No. (%)	N/A	N/A	80 (80)	39 (91)
Cumulative cisplatin dose prior to EV/LV (mg/m^2)	101 [77-119][k] n=48	100 [78-122][k] n=20	223 [163-349]	201 [159-326]
EV/LV cisplatin infusion dose, median [IQR], mg/m^2	60 [50-75]	58 [49-73]	59 [40-74]	51 [37-77]
Electrolyte supplements in 3 days prior to EV/LV, No. (%)[l]	40 (38)	14 (26)	47 (47)	17 (40)
Infection prior to EV/LV, No. (%)	16 (15)	4 (8)	36 (36)	14 (33)
PICU Admission prior to EV/LV, No. (%)	2 (2)	0 (0)	8 (8)	5 (12)
SCr-AKI episode prior to EV/LV, No. (%)[m]	13 (12)	6 (11)	46 (46)	21 (49)
eAKI episode prior to EV/LV, No. (%)	55 (52)[b]	17 (32)	93 (93)	38 (88)
Days between EV and LV visits, No. (%)	N/A	N/A	64 [42-114]	59 [41-112]
Concurrent nephrotoxins at EV/LV, No. (%)[n]	14 (13)	9 (17)	14 (14)	8 (19)
**Cancer Treatment Details**				

© 2020 McMahon KR et al. JAMA Network Open.

	EV		LV	
**Characteristic**	**eAKI (n=106)[a]**	**No eAKI (n=53)[a]**	**eAKI (n=100)[a]**	**No eAKI (n=43)[a]**
EV/LV total cisplatin cycle dose, median [IQR], mg/m^2	100 [76-121]	117 [79-194]	100 [74-120]	96 [77-144]
Any flank (left or right), whole abdomen, pelvic or total body radiation, No. (%)	15 (14)	7 (13)	11 (11)	4 (9)
**Post-Cisplatin**				
EV/LV length of stay, median [IQR], days	4 [3-14][b]	4 [2-5]	4 [3-7][b]	3 [2-4]
Nephrotoxins in 10 days post EV/LV, No. (%)[o]	11 (10)	2 (4)	7 (7)	4 (9)
Electrolyte supplements in 10 days post EV/LV, No. (%)[l]	83 (78)	38 (72)	81 (81)	32 (74)

[a]Percentages are based on the total for each column.

[b]Indicates significant difference between AKI and Non-AKI groups for that time point (p<.05).

[c]CNS Tumors: astrocytoma (n=3), choroid plexus tumor (n=2), ependymoma (n=1), medulloblastoma (n=39), primitive neuroectodermal tumor (n=7), atypical teratoid/rhabdoid tumor (n=6).

[d]Other cancers: lymphoma and nasopharyngeal carcinoma.

[e]Renal medical history: hypertension, treatment with antihypertensives, family history of kidney disease, chronic kidney disease, dialysis, congenital renal anomaly, kidney stones, vesicoureteral reflux, urinary tract infection, serum electrolyte abnormality requiring treatment or AKI.

[f]Receipt of acyclovir, amphotericin, aminoglycosides (gentamycin, tobramycin, amikacin), vancomycin, angiotensin converting enzyme inhibitor, ganciclovir/valganciclovir, ifosfamide or methotrexate in 2 weeks pre-cisplatin.

[g]GFR estimated using updated Chronic Kidney Disease in children (CKiD) equation.

[h]Pre-infusion electrolyte was determined by using the study-measured electrolyte (measured on day of cisplatin infusion, pre-infusion); if unavailable, the most recent available routine electrolyte value was used (if the study-measured electrolyte was unavailable, the routine electrolyte value from the day of cisplatin infusion, pre-infusion was used; if unavailable, the routine electrolyte value from the day before, 2 days before and 3 days before cisplatin infusion was used, respectively). n=98 for LV eAKI group for pre-infusion serum phosphorous because two individuals did not have a value available. n=41 for LV no eAKI group for pre-infusion serum magnesium because two individuals did not have a value available. To convert magnesium and phosphorus from conventional units to *Système International* (SI) units, conversion factors are 0.5 and 0.323, respectively. Multiply conventional units by conversion factor to obtain SI units (mmol/L).

[i]Denominator N is 98 instead of 100 for this variable because 2 participants did not have a pre-infusion serum phosphorous value available.

[j]Denominator N is 41 instead of 43 for this variable because 2 participants did not have a pre-infusion serum magnesium value available.

[k]No. of patients with eAKI with data is 48; No. of patients without eAKI with data is 20. These are the No. of patients that are not cisplatin naïve at EV.

[l]Any form of oral or IV supplementation of magnesium, potassium or phosphorus (excluding total parenteral nutrition).

© 2020 McMahon KR et al. JAMA Network Open.

[m]To ascertain SCr-AKI prior to EV/LV, all routine and study-measured SCr values that occurred prior to cisplatin infusion of EV/LV were used. Peak SCr was the highest SCr occurring prior to EV/LV infusion but occurring after the baseline SCr. For SCr-AKI prior to EV, baseline SCr was the lowest routine value in 3 months pre-cisplatin initiation. For AKI between EV and LV, baseline SCr was the lowest routine or study-measured value in 3 months pre-cisplatin initiation.

[n]Acyclovir, amphotericin, aminoglycosides (gentamycin, tobramycin, amikacin), ifosfamide, or chemotherapy protocol indicates a nephrotoxin (aldesleukin, busulfan, carboplatin, dinutuximab, gemcitabine, ifosfamide, lomustine, melphalan, methotrexate, radiotherapy, rituximab, stem cell transplant or temsirolimus) was given within 24h of cisplatin infusion.

[o]Acyclovir, amphotericin, aminoglycosides (gentamycin, tobramycin, amikacin), ifosfamide

Abbreviations: *CNS:* Central Nervous System; *EV*: Early Cisplatin Visit; *LV*: Late Cisplatin Visit; *AKI*: Acute Kidney Injury; *eAKI*: electrolyte-AKI; *SCr*: Serum Creatinine; *GFR*: glomerular filtration rate by nuclear medicine test clearance or 24-hour creatinine clearance; *eGFR*: estimated GFR by equation; *PICU*: pediatric intensive care unit.; *N/A*: Non applicable.

© 2020 McMahon KR et al. JAMA Network Open.

eTable 7. Characteristics of Study Population at Earlier Cisplatin Infusion Visit (EV) and Later Cisplatin Infusion Visit (LV), Comparing Patients With Versus Without AKI Defined by Having Both Serum Creatinine *and* Electrolyte-Defined AKI (SCr Plus eAKI) With Cisplatin Infusion.

	EV		LV	
**Characteristic**	**SCr plus eAKI (n=32)[a]**	**No SCr plus eAKI (n=127)[a]**	**SCr plus eAKI (n=16)[a]**	**No SCr plus eAKI (n=127)[a]**
**Baseline (Prior to 1st Cisplatin Infusion)**				
Male, No. (%)	12 (38)	68 (54)	7 (44)	62 (49)
White Race, No. (%)	27 (84)	90 (71)	13 (81)	94 (74)
Cancer diagnosis, No. (%)				
CNS Tumor[d]	14 (44)	44 (35)	7 (44)[b]	48 (38)
Neuroblastoma	10 (31)	33 (26)	6 (38)	25 (20)
Osteosarcoma	2 (6)	31 (24)	0 (0)	33 (26)
Germ cell tumor	3 (9)	11 (9)	0 (0)	14 (11)
Hepatoblastoma	3 (9)	6 (5)	2 (13)	7 (6)
Other[e]	0 (0)	2 (2)	1 (6)	0 (0)
Cancer involves one or both kidneys, No. (%)	3 (9)	8 (6)	2 (13)	5 (4)
Renal medical history, No. (%)[f]	7 (22)[b]	6 (5)	0 (0)	11 (9)
Any nephrotoxic drug prior to 1st cisplatin, No. (%)[g]	8 (25)	19 (15)	N/A	N/A
Vancomycin in 2 weeks pre-cisplatin, No. (%)	4 (13)	4 (3)	N/A	N/A
**Immediately Prior to/Day of EV/LV Infusion**				
Age at EV/LV, median [IQR], years	2.7 [2.2-8.3][b]	6.3 [2.8-12.3]	3.2 [2.4-4.9]	6.7 [2.5-13.6]
Age at EV/LV <3 years, No. (%)	17 (53)[b]	35 (28)	7 (44)	38 (30)
Cisplatin naïve at EV	20 (63)	71 (56)	N/A	N/A
Pre-visit eGFR, median [IQR], $mL/min/1.73m^2$ [h]	166 [145-206][c]	135 [117-160]	163 [152-192][b]	134 [117-169]
Pre-infusion serum phosphorus, mean (SD), mg/dL[i]	4.4 (1.1)	4.7 (1.2)	4.1 (0.8)[b]	4.8 (0.9)[j] n=125
Pre-infusion serum magnesium, mean (SD), mEq/L[i]	1.5 (0.3)[b]	1.6 (0.2)	1.4 (0.1)[b]	1.6 (0.2)[j] n=125
LV was the last cisplatin cycle of cancer treatment, No. (%)	N/A	N/A	12 (75)	107 (84)
Cumulative cisplatin dose prior to EV/LV, median [IQR], mg/m^2	93 [79-135][k] n=12	104 [77-120][k] n=56	183 [149-202]	223 [165-349]
EV/LV cisplatin infusion dose, median [IQR], mg/m^2	72 [44-77]	59 [49-74]	70 [50-78]	58 [37-74]
Electrolyte supplements in 3 days prior to EV/LV, No. (%)[l]	14 (44)	40 (32)	8 (50)	56 (44)
Infection prior to EV/LV, No. (%)	5 (16)	15 (12)	9 (56)	41 (32)
PICU Admission prior to EV/LV, No. (%)	1 (3)	1 (1)	3 (19)	10 (8)

© 2020 McMahon KR et al. JAMA Network Open.

	EV		LV	
**Characteristic**	**SCr plus eAKI (n=32)[a]**	**No SCr plus eAKI (n=127)[a]**	**SCr plus eAKI (n=16)[a]**	**No SCr plus eAKI (n=127)[a]**
SCr-AKI episode prior to EV/LV, No. (%)[m]	6 (19)	13 (10)	11 (69)	56 (44)
eAKI episode prior to EV/LV, No. (%)	19 (59)	53 (42)	15 (94)	116 (91)
Days between EV and LV visits, median [IQR]	N/A	N/A	43 [34-71]	64 [42-117]
Concurrent nephrotoxins at EV/LV, No. (%)[n]	3 (9)	20 (16)	2 (13)	20 (16)
**Cancer Treatment Details**				
EV/LV total cisplatin cycle dose, median [IQR], mg/m^2	92 [76-137]	109 [78-143]	84 [75-197]	100 [75-120]
Any flank (left or right), whole abdomen, pelvic or total body radiation, No. (%)	6 (19)	16 (13)	3 (19)	12 (9)
**Post-Cisplatin**				
EV/LV length of stay, median [IQR], days	7 [4-33][b]	4 [3-5]	5 [3-15][b]	4 [2-5]
Nephrotoxins in 10 days post EV/LV, No. (%)[o]	5 (16)	8 (6)	1 (6)	10 (8)
Electrolyte supplements in 10 days post EV/LV, No. (%)[l]	27 (84)	94 (74)	10 (63)	103 (81)

[a]Percentages are based on the total for each column.

[b]Indicates significant difference between AKI and Non-AKI groups for that time point (p<.05).

[c]Indicates significant difference between AKI and Non-AKI groups for that time point (p<.001).

[d]CNS Tumors: astrocytoma (n=3), choroid plexus tumor (n=2), ependymoma (n=1), medulloblastoma (n=39), primitive neuroectodermal tumor (n=7), atypical teratoid/rhabdoid tumor (n=6).

[e]Other cancers: lymphoma and nasopharyngeal carcinoma.

[f]Renal medical history: hypertension, treatment with antihypertensives, family history of kidney disease, chronic kidney disease, dialysis, congenital renal anomaly, kidney stones, vesicoureteral reflux, urinary tract infection, serum electrolyte abnormality requiring treatment or AKI.

[g]Receipt of acyclovir, amphotericin, aminoglycosides (gentamycin, tobramycin, amikacin), vancomycin, angiotensin converting enzyme inhibitor, ganciclovir/valganciclovir, ifosfamide or methotrexate in 2 weeks pre-cisplatin.

[h]GFR estimated using updated Chronic Kidney Disease in children (CKiD) equation.

iPre-infusion electrolyte was determined by using the study-measured electrolyte (measured on day of cisplatin infusion, pre-infusion); if unavailable, the most recent available routine electrolyte value was used (if the study-measured electrolyte was unavailable, the routine electrolyte value from the day of cisplatin infusion, pre-infusion was used; if unavailable, the routine electrolyte value from the day before, 2 days before and 3 days before cisplatin infusion was used, respectively). n=125 for LV non-AKI group because two individuals did not have a pre-infusion serum phosphorous and magnesium value available. To convert magnesium and phosphorus from conventional units to *Système International* (SI) units, conversion factors are 0.5 and 0.323, respectively. Multiply conventional units by conversion factor to obtain SI units (mmol/L).

© 2020 McMahon KR et al. JAMA Network Open.

[j]Denominator N is 125 instead of 127 for this variable because 2 participants did not have a pre-infusion electrolyte value available.

[k]No. of patients with SCr plus eAKI with data is 12; No. of patients without SC plus eAKI with data is 56. These are the No. of patients that are not cisplatin naïve at EV.

[l]Any form of oral or IV supplementation of magnesium, potassium or phosphorus (excluding total parenteral nutrition).

[m]To ascertain SCr-AKI prior to EV/LV, all routine and study-measured SCr values that occurred prior to cisplatin infusion of EV/LV were used. Peak SCr was the highest SCr occurring prior to EV/LV infusion but occurring after the baseline SCr. For SCr-AKI prior to EV, baseline SCr was the lowest routine value in 3 months pre-cisplatin initiation. For AKI between EV and LV, baseline SCr was the lowest routine or study-measured value in 3 months pre-cisplatin initiation.

[n]Acyclovir, amphotericin, aminoglycosides (gentamycin, tobramycin, amikacin), ifosfamide, or chemotherapy protocol indicates a nephrotoxin (aldesleukin, busulfan, carboplatin, dinutuximab, gemcitabine, ifosfamide, lomustine, melphalan, methotrexate, radiotherapy, rituximab, stem cell transplant or temsirolimus) was given within 24h of cisplatin infusion.

[o]Acyclovir, amphotericin, aminoglycosides (gentamycin, tobramycin, amikacin), ifosfamide

Abbreviations: *CNS:* Central Nervous System; *EV*: Early Cisplatin Visit; *LV*: Late Cisplatin Visit; *AKI*: Acute Kidney Injury; *eAKI*: electrolyte-AKI; *SCr*: Serum Creatinine; *GFR*: glomerular filtration rate by nuclear medicine test clearance or 24-hour creatinine clearance; *eGFR*: estimated GFR by equation; *PICU*: pediatric intensive care unit; *N/A*: Non applicable.

© 2020 McMahon KR et al. JAMA Network Open.

eTable 8. Fractional Excretion of Electrolytes at Earlier Cisplatin Infusion Visit (EV) and Later Cisplatin Infusion Visit (LV).

EV[a]			
	**Pre-Infusion (n=149)[b, c]**	**Post-Infusion (n=147)[d, e]**	**Discharge (n=144)[f, g]**
FE(Mg), median [IQR], %	3.5 [2.5-5.3]	9.0 [5.0-14.4][h]	8.0 [4.3-14.1][h]
	eAKI: 3.7 [2.5-5.5]	eAKI: 9.4 [5.2-14.8]	eAKI: 7.9 [4.4-12.7]
	No eAKI: 3.0 [2.4-5.0]	No eAKI: 8.3 [5.0-11.6]	No eAKI: 9.0 [4.1-15.0]
FE(K), median [IQR], %	9.1 [6.4-14.7]	12.3 [7.5-17.5][h]	11.0 [8.2-16.8][i]
	eAKI: 9.7 [6.6-13.9]	eAKI: 11.7 [8.0-17.0]	eAKI: 11.0 [7.9-16.7]
	No eAKI: 8.3 [6.3-17.1]	No eAKI: 13.6 [7.1-19.8]	No eAKI: 11.0 [8.8-17.0]
FE(P), median [IQR], %	7.4 [5.1-11.0][j]	12.1 [7.3-16.5][h]	15.1 [9.5-20.7][h, k]
	eAKI: 7.2 [5.0-11.0][k]	eAKI: 12.6 [7.7-16.6]	eAKI: 15.9 [10.0-21.7]
	No eAKI: 7.6 [5.2-11.1]	No eAKI: 9.3 [7.2-14.4]	No eAKI: 14.0 [8.3-20.3]
**LV[a]**			
	**Pre-Infusion (n=120)[b, m]**	**Post-Infusion (n=122)[d, n]**	**Discharge (n=115)[f, o]**
FE(Mg), median [IQR], %	4.1 [2.6-6.7]	9.1 [4.8-14.3][h]	9.3 [5.6-15.2][h, p]
	eAKI: 4.1 [2.7-6.7]	eAKI: 9.4 [4.8-14.3]	eAKI: 8.2 [5.6-15.1]
	No eAKI: 3.8 [2.5-6.6]	No eAKI: 8.6 [4.9-14.1]	No eAKI: 10.2 [7.4-15.5]
FE(K), median [IQR], %	10.3 [6.4-14.6]	11.2 [7.9-17.0][i]	11.7 [7.9-16.2][i]
	eAKI: 9.8 [6.3-13.5]	eAKI: 11.5 [7.7-18.3]	eAKI: 11.5 [6.9-16.7]
	No eAKI: 12.0 [6.5-16.1]	No eAKI: 10.2 [8.0-15.9]	No eAKI: 12.0 [9.2-14.9]
FE(P), median [IQR], %	7.0 [5.3-10.6]	11.0 [7.7-16.5][h]	12.2 [7.4-19.7][h, p]
	eAKI: 7.5 [5.3-10.9]	eAKI: 11.5 [8.2-17.7]	eAKI: 13.3 [7.5-21.3]
	No eAKI: 6.8 [5.1-9.4]	No eAKI: 9.6 [5.0-12.2]	No eAKI: 10.2 [6.1-15.8]

[a]Total sample size was not available to measure both serum and urine creatinine and electrolytes at EV and LV due to sample miscollection or insufficient quantity for analyte measurements (eTable 3).

© 2020 McMahon KR et al. JAMA Network Open.

[b]Pre (pre-infusion) serum collected on the day of the infusion before infusion start.

[c]EV pre-infusion: No eAKI group n=48, eAKI n=101 (n=100 for phosphorus due to insufficient quantity of urine).

[d]Post (post-infusion) serum collected the morning after the cisplatin infusion.

[e]EV post-infusion: No eAKI group n=47, eAKI n=100.

[f]Discharge serum collected just before discharge from hospital (day 2 to 5).

[g]EV discharge: No eAKI group n=47, eAKI n=97.

[h]Indicates significant difference from pre-infusion time point by Wilcoxon signed-rank test (P<.001).

[i]Indicates significant difference from pre-infusion time point by Wilcoxon signed-rank test (p<.05).

[j]The number of participants with pre-infusion FE(P) at EV is 148 due to insufficient quantity of urine.

[k]The number of participants with eAKI with pre-infusion FE(P) at EV is 100 for phosphorus due to insufficient quantity of urine.

[l]Indicates significant difference from post-infusion time point by Wilcoxon signed-rank test (p<.001).

[m]LV pre-infusion: No eAKI group n=36, eAKI n=84.

[n]LV post-infusion: No eAKI group n=37, eAKI n=85.

[o]LV discharge: No eAKI group n=32, eAKI n=83.

[p]Indicates significant difference from post-infusion time point by Wilcoxon signed-rank test (p<.05).

Abbreviations: *EV*: Early Cisplatin Visit; *LV*: Late Cisplatin Visit; *FE*: Fractional Excretion; *P*: Phosphorus; *Mg*: Magnesium; *K*: Potassium; *Discharge*: hospital discharge.

© 2020 McMahon KR et al. JAMA Network Open.

eTable 9. Characteristics of Study Participants at EV and LV Stratified by Cancer Type.

EV (n=159)						
**EV Characteristics**	**CNS Tumor[a] (n=58)**	**Neuroblastoma (n=43)**	**Osteosarcoma (n=33)**	**Germ Cell Tumor (n=14)**	**Hepatoblastoma (n=9)**	**Other[b] (n=2)**
Age at EV, median [IQR], years	5.8 [2.3-9.2]	3.8 [2.0-4.8]	13.4 [9.8-15.0]	13.3 [1.8-16.2]	2.0 [1.6-2.8]	11.7 [11.5-11.8]
Male, No. (%)	31 (53)	25 (58)	14 (42)	3 (21)	5 (56)	2 (100)
EV Cisplatin Infusion Dose, median [IQR], mg/m^2	75 [73-79]	49 [39-50]	59 [59-60]	20 [20-24]	80 [70-81]	78 [75-80]
SCr-AKI, No. (%)	20 (34)	18 (42)	2 (6)	3 (21)	4 (44)	1 (50)
**LV (n=143)**						
**LV Characteristics**	**CNS Tumor[c] (n=55)**	**Neuroblastoma (n=31)**	**Osteosarcoma (n=33)**	**Germ Cell Tumor (n=14)**	**Hepatoblastoma (n=9)**	**Other[d] (n=1)**
Age at LV, median [IQR], years	6.1 [2.4-10.2]	3.2 [1.9-4.6]	13.7 [10.0-15.2]	13.4 [1.9-16.3]	2.3 [1.8-2.9]	11.9 [11.9-11.9]
Male, No. (%)	29 (53)	17 (55)	14 (42)	3 (21)	5 (56)	1 (100)
LV Cisplatin Infusion Dose, median [IQR], mg/m^2	75 [38-80]	49 [37-51]	59 [58-60]	20 [19-24]	80 [70-83]	76 [76-76]
SCr-AKI, No. (%)	11 (20)	8 (26)	0 (0)	0 (0)	3 (33)	1 (100)

[a]CNS Tumor: astrocytoma (n=3), choroid plexus tumor (n=2), ependymoma (n=1), medulloblastoma (n=39), primitive neuroectodermal tumor (n=7), atypical teratoid/rhabdoid tumor (n=6).

[b]Other cancers: lymphoma and nasopharyngeal carcinoma.

[c]CNS Tumor: astrocytoma (n=2), choroid plexus tumor (n=2), ependymoma (n=1), medulloblastoma (n=39), primitive neuroectodermal tumor (n=7), atypical teratoid/rhabdoid tumor (n=4).

[d]Other cancer: nasopharyngeal carcinoma.

Abbreviations: *CNS:* Central Nervous System; *EV*: Early Cisplatin Visit; *LV*: Late Cisplatin Visit; *SCr*: Serum Creatinine; *AKI*: Acute Kidney Injury.

© 2020 McMahon KR et al. JAMA Network Open.

eTable 10: Cancer Treatment Details at Earlier Cisplatin Infusion Visit (EV) and Later Cisplatin Infusion Visit (LV) Stratified by Cancer Type.

EV									
**Cancer Diagnosis**	**Total Number of Cisplatin Infusions Received up until EV (including EV infusion), median [IQR]**	**Total Cisplatin Dose Received prior to EV Cycle (excludes EV cycle), median [IQR], mg/m^2**	**Total Number of Cisplatin Infusions Received at Current EV Cycle, median [IQR]**	**Dose of First Cisplatin Infusion of EV Cycle, median [IQR], mg/m^2**	**Total Cisplatin Dose of EV Cycle, median [IQR], mg/m^2**	**Total Number of Other Nephro-toxins or Nephrotoxic Treatments in Protocol, median [IQR][a]**	**Cyclophos-phamide in protocol, No. (%)**	**Etoposide in protocol, No. (%)**	**Vincristine in Protocol, No. (%)**
CNS Tumors (n=58)	2 [1-2]	77 [73-83] (n=30)	1 [1-1]	75 [73-79]	75 [73-79]	2 [2-2]	51 (88)	29 (50)	58 (100)
Germ Cell Tumour (n=14)	1 [1-1]	94 [62-105] (n=3)	5 [3-5]	20 [20-24]	100 [94-101]	0 [0-0]	2 (14)	14 (100)	0 (0)
Hepatoblastoma (n=9)	1 [1-2]	82 [81-90] (n=4)	1 [1-1]	80 [70-81]	80 [70-81]	1 [0-1]	0 (0)	0 (0)	4 (44)
Neuroblastoma (n=43)	1 [1-1]	201 [156-207] (n=7)	4 [4-4]	49 [39-50]	198 [157-202]	4 [4-4]	43 (100)	43 (100)	43 (100)
Osteosarcoma (n=33)	3 [1-3	119 [118-121] (n=24)	2 [2-2]	59 [59-60]	118 [118-120]	1 [1-1]	0 (0)	7 (21)	0 (0)
Other cancers (n=2)	1 [1-1]	N/A (n=0)	1 [1-1]	78 [75-80]	78 [75-80]	3 [1-4]	0 (0)	1 (50)	0 (0)
Total (n=159)	*1 [1-2]*	*101 [77-120] (n=68)*	*2 [1-4]*	*59 [49-75]*	*102 [77-138]*	*2 [1-4]*	*96 (60)*	*94 (59)*	*105 (66)*

© 2020 McMahon KR et al. JAMA Network Open.

LV									
**Cancer Diagnosis**	**Total Number of Cisplatin Infusions Received up until LV (including LV infusion), median [IQR]**	**Total Cisplatin Dose Received prior to LV Cycle (excludes LV cycle), median [IQR], mg/m^2**	**Total Number of Cisplatin Infusions Received at Current LV Cycle, median [IQR]**	**Dose of First Cisplatin Infusion of LV Cycle, median [IQR], mg/m^2**	**Total Cisplatin Dose of LV Cycle, median [IQR], mg/m^2**	**Total Number of Other Nephro-toxins or Nephrotoxic Treatments in Protocol, median [IQR][a]**	**Cyclophos-phamide in protocol, No. (%)**	**Etoposide in protocol, No. (%)**	**Vincristine in Protocol, No. (%)**
CNS Tumors (n=55)	4 [3-6]	213 [159-327]	1 [1-1]	75 [38-80]	75 [40-80]	2 [2-2]	48 (87)	26 (47)	55 (100)
Germ Cell Tumour (n=14)	11 [6-16]	204 [102-301]	5 [3-5]	20 [19-24]	98 [78-100]	0 [0-0]	2 (14)	14 (100)	0 (0)
Hepatoblastoma (n=9)	5 [3-6]	203 [168-362]	1 [1-1]	80 [70-83]	80 [70-83]	1 [0-1]	0 (0)	0 (0)	4 (44)
Neuroblastoma (n=31)	5 [5-5]	198 [156-202]	4 [4-4]	49 [37-51]	197 [148-202]	4 [4-5]	31 (100)	31 (100)	31 (100)
Osteosarcoma (n=33)	7 [5-7]	353 [241-359]	2 [2-2]	59 [58-60]	118 [117-120]	1 [1-1]	0 (0)	7 (21)	0 (0)
Other cancer (n=1)	3 [3-3]	157 [157-157]	1 [1-1]	76 [76-76]	76 [76-76]	1 [1-1]	0 (0)	1 (100)	0 (0)
Total (n=143)	*5 [4-6]*	*208 [161-339]*	*2 [1-4]*	*58 [37-74]*	*98 [75-120]*	*2 [1-3]*	*81 (57)*	*78 (55)*	*90 (63)*

[a]Each of the following drugs or treatments were considered if they were included in the chemotherapy protocol: aldesleukin, busulfan, carboplatin, dinutuximab, gemcitabine, ifosfamide, lomustine, melphalan, methotrexate, radiotherapy, rituximab, stem cell transplant or temsirolimus.

Abbreviations: *CNS:* Central Nervous System; *EV*: Early Cisplatin Visit; *LV*: Late Cisplatin Visit.

© 2020 McMahon KR et al. JAMA Network Open.
